# Dual role of lipids for genome stability and pluripotency facilitates full potency of mouse embryonic stem cells

**DOI:** 10.1093/procel/pwad008

**Published:** 2023-02-16

**Authors:** Liangwen Zhong, Miriam Gordillo, Xingyi Wang, Yiren Qin, Yuanyuan Huang, Alexey Soshnev, Ritu Kumar, Gouri Nanjangud, Daylon James, C David Allis, Todd Evans, Bryce Carey, Duancheng Wen

**Affiliations:** Department of Reproductive Medicine, Ronald O. Perelman and Claudia Cohen Center for Reproductive Medicine, Weill Cornell Medicine, New York, NY 10065, USA; Department of Surgery, Weill Cornell Medical College, 1300 York Avenue, New York, NY 10065, USA; Cold Spring Harbor Laboratory, Cold Spring Harbor, NY 11724, USA; Department of Reproductive Medicine, Ronald O. Perelman and Claudia Cohen Center for Reproductive Medicine, Weill Cornell Medicine, New York, NY 10065, USA; Department of Reproductive Medicine, Ronald O. Perelman and Claudia Cohen Center for Reproductive Medicine, Weill Cornell Medicine, New York, NY 10065, USA; Laboratory of Chromatin Biology and Epigenetics, The Rockefeller University, New York, NY 10065, USA; Department of Neuroscience, Developmental and Regenerative Biology, University of Texas at San Antonio, One UTSA Circle, San Antonio, TX 78249, USA; Department of Surgery, Weill Cornell Medical College, 1300 York Avenue, New York, NY 10065, USA; Gladstone Institutes, 1650 Owens St, San Francisco, CA 94158, USA; Molecular Cytogenetics Core. Memorial Sloan Kettering Cancer Center, New York, NY 10065, USA; Department of Reproductive Medicine, Ronald O. Perelman and Claudia Cohen Center for Reproductive Medicine, Weill Cornell Medicine, New York, NY 10065, USA; Laboratory of Chromatin Biology and Epigenetics, The Rockefeller University, New York, NY 10065, USA; Department of Surgery, Weill Cornell Medical College, 1300 York Avenue, New York, NY 10065, USA; Laboratory of Chromatin Biology and Epigenetics, The Rockefeller University, New York, NY 10065, USA; Department of Reproductive Medicine, Ronald O. Perelman and Claudia Cohen Center for Reproductive Medicine, Weill Cornell Medicine, New York, NY 10065, USA

**Keywords:** mouse pluripotent stem cells, lipids, pluripotency transition, genomic stability, developmental potency, nucleotide pool depletion, 2i medium, X chromosome loss, female all-ESC mice

## Abstract

While Mek1/2 and Gsk3β inhibition (“2i”) supports the maintenance of murine embryonic stem cells (ESCs) in a homogenous naïve state, prolonged culture in 2i results in aneuploidy and DNA hypomethylation that impairs developmental potential. Additionally, 2i fails to support derivation and culture of fully potent female ESCs. Here we find that mouse ESCs cultured in 2i/LIF supplemented with lipid-rich albumin (AlbuMAX) undergo pluripotency transition yet maintain genomic stability and full potency over long-term culture. Mechanistically, lipids in AlbuMAX impact intracellular metabolism including nucleotide biosynthesis, lipid biogenesis, and TCA cycle intermediates, with enhanced expression of DNMT3s that prevent DNA hypomethylation. Lipids induce a formative-like pluripotent state through direct stimulation of Erk2 phosphorylation, which also alleviates X chromosome loss in female ESCs. Importantly, both male and female “all-ESC” mice can be generated from *de novo* derived ESCs using AlbuMAX-based media. Our findings underscore the importance of lipids to pluripotency and link nutrient cues to genome integrity in early development.

## Introduction

Murine embryonic stem cells (ESCs) have unlimited self-renewal and differentiation capacity (Evans and Kaufman, 1981; Martin, 1981), as demonstrated by their ability to generate all ESC-derived mice (all-ESC mice) with the help of tetraploid (4n) embryos (Nagy et al., 1993; Eggan et al., 2001; George et al., 2007; Wen et al., 2014). Murine ESCs were initially derived in media supplemented with fetal bovine serum (FBS) on feeder layers of mouse embryonic fibroblasts (Evans and Kaufman, 1981; Martin, 1981). Yet undefined serum components results in heterogeneous cultures and gradual loss of pluripotency (Hackett and Surani, 2014). The breakthrough discovery that inhibition of Mek1/2 and Gsk3β (2i) supplemented with leukemia inhibitory factor (LIF) maintains murine ESCs in a more homogenous ground state (Ying et al., 2008), enabled derivation and expansion of ESCs from various mouse backgrounds including non-permissive strains. However, prolonged Mek1/2 suppression of murine ESCs in 2i/LIF medium results in aneuploidy, DNA hypomethylation, and loss of imprinting, thus impairs developmental potential, which is typically more prominent for female ESCs leading to rapid loss of pluripotency in early passages (Choi et al., 2017; Yagi et al., 2017). The alternative 2i/LIF (Mek1/2 inhibitor is replaced by Src inhibitor, a2i/LIF) can improve the epigenetic integrity and developmental potential of mouse ESCs up to passage 15 (Shimizu et al., 2012; Choi et al., 2017; Yagi et al., 2017; Zhang et al., 2022), however, it still does not support the culture of female ESCs to generate adult all-ESC mice (Choi et al., 2017; Yagi et al., 2017). These findings indicate that naïve ESCs induced by Mek1/2 suppression cannot be maintained long-term *in vitro*, suggesting that a new approach is needed for deriving and maintaining mouse ESCs with full developmental potential.

Lipids play vital roles in the maintenance of cellular homeostasis by serving as an energy source through mitochondrial fatty acid oxidation, enhancing intracellular signal transduction, and providing macromolecules for membrane biosynthesis during growth and proliferation (Tsogtbaatar et al., 2020). Lipids also affect stem cell pluripotency as demonstrated in recent reports that lipid supplementation in E8 medium induces differentiation of intermediate human ESCs into a primed stage (Cornacchia et al., 2019; Xu et al., 2021) and regulate human ESCs self-renewal (Garcia-Gonzalo and Belmonte, 2008).

Here, we report that supplementing AlbuMAX (AX, lipids rich albumin) in 2i/LIF medium significantly improves genomic stability and developmental potency of murine ESCs over long-term culture. Both male and female all-ESC mice are generated from *de novo* derived ESCs using AX-based medium. We provide definitive evidence that lipids maintain genomic stability and drive pluripotency transition, via impacting ESCs intracellular metabolism including nucleotide biosynthesis pathways and induce a formative-like pluripotency state mediated through Erk signaling.

## Results

### AX improves developmental potential of murine ESCs

**Figure 1.**
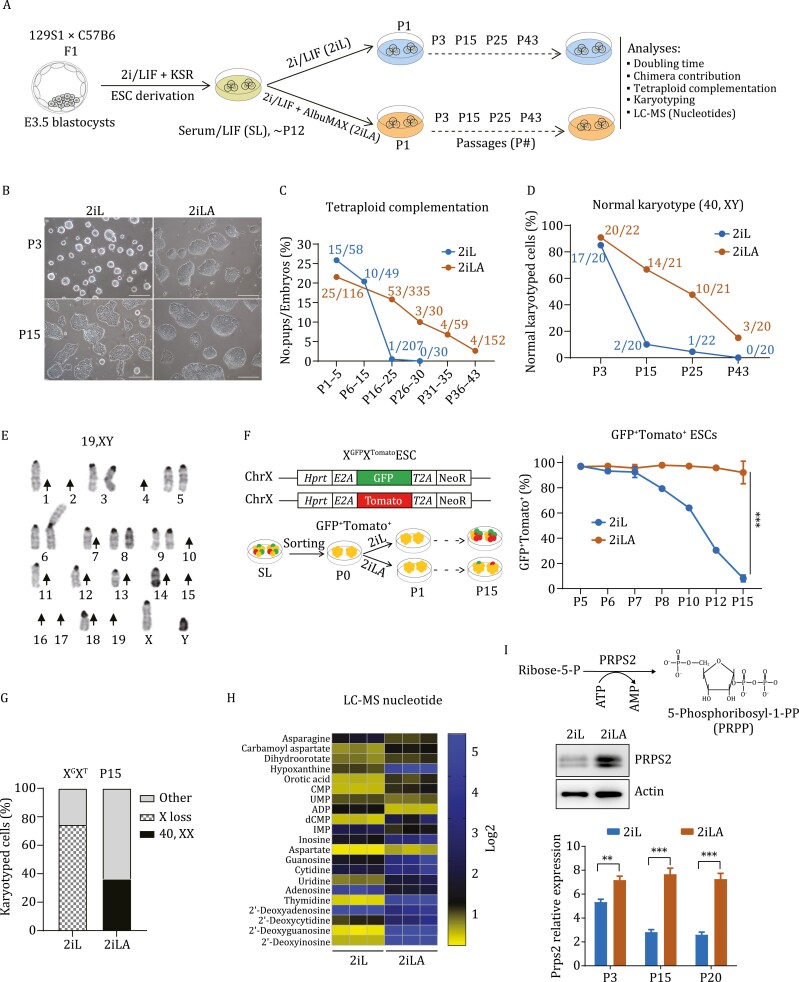
**AX improves developmental potential and genomic stability of murine ESCs.** (A) Schematic illustration of the experimental design. (B) Colony morphology for ESCs cultured in 2iL or 2iLA at passage 3 or passage 15. Scale, 25 µm. (C) Developmental potential assessed by tetraploid complementation. The numbers at each timepoint represent total No. pups/No. embryos transferred. (D and E) Analysis for normal karyotype (D) in 2iL and 2iLA at different passages (*n* = ~20 metaphases at each passage for 2iL or 2iLA). Representative severe chromosome loss (E) in 2iL at P43. Arrow: Chromosome loss. Note both sister chromosomes were lost for chromosome 2, 4, 10, 15, 16, 17, 19 without any trisomy in this spread. (F) FACS analysis of X^G^X^T^ reporter female ESC cultured in 2iL or 2iLA. The double reporter positive cells indicate both X chromosomes are maintained. ^***^P < 0.001, two-tailed *t*-test. (G) Karyotyping of the XGXT ESCs cultured in 2iL and 2iLA at passage 15. (H) LC–MS analysis of nucleotide pools for ESCs cultured in 2iL and 2iLA (P3). (I) Prps2 expression levels by qRT-PCR assay or Western blot assay. ^**^P < 0.01, ^***^P < 0.001, multiple unpaired T-test.

Murine naïve stem cells are maintained in a serum-free medium supplemented with GSK3β and Mek inhibitors (PD184352 and CHIR99021, 2i) and leukemia inhibitory factor (LIF) (Ying et al., 2008) (hereafter 2iL for the 2i/LIF medium or 2i-ESCs for the ESCs cultured in 2i/LIF medium). To test how lipids can influence pluripotent stem cells in serum-free media conditions, a chromatographically purified lipid-rich bovine serum albumin, AlbuMAX (A or AX), was added to 2iL culture medium (hereafter 2iLA for 2i/LIF+ AlbuMAX medium or AX-ESCs for ESCs cultured in 2iLA medium) (Fig. 1A). Male ESCs cultured in 2iLA medium maintained bright but flatter/larger colonies for over 15 passages, whereas the colonies in 2iL medium without AX acquired a flat and dark appearance (Figs. 1B and S1A). ESCs cultured in 2iLA proliferated more rapidly than in 2iL with doubling time at early passages (p3–5) of 6.7 h compared to 10.2 h, respectively (Fig. S1A). Over continued passaging, the proliferation of AX-ESCs gradually slowed with doubling time increasing to 8.1 h at passage 15 and reaching to 10.7 h at passage 20 (Fig. S1A).

After injection into blastocyst embryos, AX-ESCs gave rise to post-natal chimeras with germline transmission (Fig. S1B). To test for full developmental potency, AX-ESCs were assayed for the ability to generate all-ESC mice by tetraploid complementation. Both 2i-ESCs and AX-ESCs could generate all-ESC mice at high efficiency in early passages (p1–5) (Fig.1C). Consistent with previous studies (Choi et al., 2017; Yagi et al., 2017), prolonged culture of ESCs in 2iL demonstrated loss of developmental potential by 15 passages. Intriguingly, ESCs cultured in 2iLA medium retained the potential to generate all-ESC mice for over 20 passages (Figs. 1C, S1C and S1D). We frequently obtained as many as seven full-term live pups from a single surrogate mother (Figs. 1C and S1C), efficiency similar to early passage 2i-ESCs. Although the potential for AX-ESCs to generate all-ESC mice decreased over time, it extended to passage 43 after 3 months of culture (Figs. 1C and S1E).

### AX promotes karyotypic stability in ESC cultures

Because aneuploidy is the leading genetic cause of early pregnancy loss (Yuan et al., 2002), we speculated that improved developmental potential of AX-ESCs may be due to improved genomic stability. We analyzed the karyotypes of ESCs cultured in 2iL and 2iLA media. Over 85% of ESCs cultured in either 2iL or 2iLA showed normal karyotype (40, XY) at passage 3 (P3), but by passage 15–25, nearly 95% of the cells maintained in 2iL exhibited aneuploidy (Fig. 1D; Table S1), with trisomy 8 prevailing among aneuploid cells (Fig. S2A–C). By passage 43 nearly 100% of cells displayed aneuploidy, with a high prevalence of chromosome loss (Figs. 1D, 1E, S2D and S2E; Table S1). Many of these ESCs had reduced chromosome content that approached haploid karyotype (Figs. 1E, S2D, S2E; Table S1). Remarkably, ESCs cultured in 2iLA medium had significantly higher number of cells exhibiting a normal karyotype, with over 68.3%, 50%, and 16.7% at passages 15, 25, and 43, respectively (Fig. 1D; Table S1) indicating increased genome stability in cells grown in 2iLA. In addition, unlike 2i-ESCs, AX-ESCs maintained a near diploid karyotype (Fig. S2D and S2E). Female ESCs tend to quickly loss one of their two X chromosomes in 2iL medium (Choi et al., 2017; Yagi et al., 2017), so a dual reporter female ESC line (X^GFP^X^Tomato^, X^G^X^T^) (Choi et al., 2017) was employed to measure X chromosome loss by flow cytometry. Consistent with increased genomic instability, approximately 40% of cells at passage 10, and over 90% at passage 15, lost one X chromosome in ESCs cultured in 2iL medium (Fig. 1F). Notably, ESCs in 2iLA medium maintained two X chromosomes in over 95% of the cells with very little fluctuation from passage 3 to passage 15 (Fig. 1F). Karyotyping of the X^G^X^T^ ESCs at P15 confirmed the physical loss of X chromosome in 2i-ESCs and thus excluded the possibility of X chromosome inactivation in 2iL medium (Fig. 1G; Table S1). Together, these data suggest that AX can efficiently prevent X chromosome loss in female ESCs and promotes karyotypic stability in ESC cultures.

The extensively reduced chromosome content, typically loss of both pair of autosomal chromosomes in late passages of 2i-ESCs (Figs. 1E, S2D and S2E; Table S1), indicates a possible deficiency in DNA replication and repair, which could be associated with imbalanced endogenous nucleotide pools (Magdalou et al., 2014; Fasullo and Endres, 2015; Pai and Kearsey, 2017). We performed liquid chromatography–mass spectrometry (LC–MS) comparing ESCs cultured in 2iL or 2iLA conditions. Steady-state endogenous nucleotide pools are severely depleted in 2i-ESCs compared to AX-ESCs (Fig. 1H). Most of the nucleotides including thymidine, 2ʹ-deoxyguanosine, 2ʹ-deoxyinosine, and the precursor aspartate, are dramatically reduced in 2i-ESCs. Speculating that nucleotide biosynthesis pathways are impacted in 2iL medium, resulting in depletion of endogenous nucleotide pools, we analyzed the expression of Prps2, an ATP-dependent enzyme in the syntheses of purines and pyrimidines from ribose 5-phosphate (Visentin et al., 1977; Li et al., 2007). Real-time quantitative RT-PCR (qPCR) and Western blot analyses showed that Prps2 is expressed at significantly lower levels in 2i-ESCs compared to AX-ESCs (Figs. 1I and S2F; Table S1), supporting the hypothesis that nucleotide biosynthesis is attenuated in 2iL medium and maintained by AX supplementation. We added exogenous nucleoside (ES-008-D, Millipore Sigma) into 2i/LIF medium, and did observe improvement of colony morphology over passaging compared to 2iL ESCs (Fig. S2G), but not a rescue of aneuploidy based on karyotyping at P15 (Fig. S2H). Indeed, trisomy 6, 8 still contributed to over 65% of the aneuploid cells (Fig. S2H), indicating that adding nucleosides alone in 2i/LIF is not sufficient to alleviate the occurrence of trisomy in 2i-ESCs.

### AX induces the transition of ESCs from naïve to formative-like pluripotency

**Figure 2.**
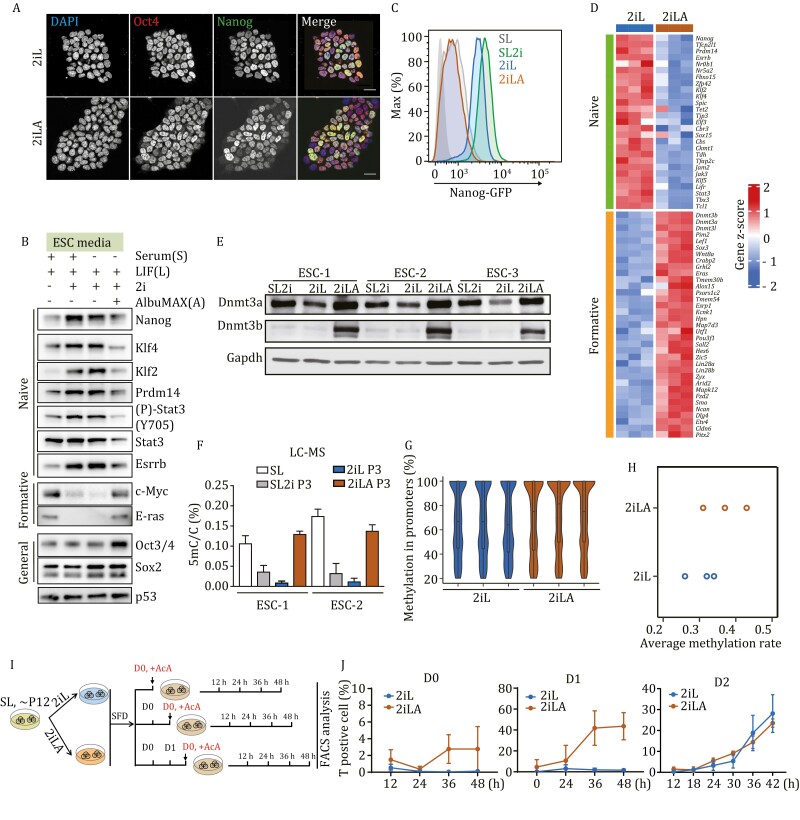
**AX induces the transition of ESCs from naive to formative-like pluripotency.** (A) Immunostaining for Oct4 (red) and Nanog (green) of ESCs cultured in 2iL or 2iLA. DAPI (Blue) for cell nuclei. (B) Western blot of the naive, formative or general pluripotency marker protein level in SL, SL2i, 2iL or 2iLA. (C) FACS analysis using a GFP:Nanog ESC reporter line, the GFP fluorescence intensity is decreased in 2iLA. (D) Heatmap of transcript levels from bulk RNA-seq for the naive and formative genes cultured in 2iL or 2iLA at P3. Three different ESC lines for biological replicates. (E) Dnmt3a/b levels demonstrated by Western blot assays. (F) LC–MS showing the global levels of 5mC/C ratio in genomic DNA from ESCs cultured in different media. (G) Violin plots of the DNA methylation distribution rate at promoter regions in ESCs cultured in 2iL or 2iLA at P3 from reduced-representation bisulfite sequencing (RRBS). Three biological replicates. (H) RRBS for quantification of the average global methylation rate in all CpGs. Three biological replicates. (I) Schematic of the assay to test responsiveness to Activin A for differentiation. (J) FACS analysis of the mesendoderm differentiation marker brachyury (T) positive cells at different time points after Activin A (AcA) addition at Day 0 (D0), Day 1 (D1) or Day 2 (D2) embryoid body formation. D0, D2 two biological replicates, D1 three biological replicates.

While AX-ESCs retained the potential to generate all-ESC mice (Fig. 1C), we observed that colonies of these ESCs no longer maintained the typical uniform and rounded naïve colony morphology. Analyses of the core pluripotency genes, Pou5f1 (Oct3/4) and Sox2 by immunostaining, qPCR and Western blot assays revealed that these genes are expressed at similar levels independent of culture medium (Figs. 2A, 2B and S3A). However, genes considered as naïve markers, such as Nanog, Rex1 (Zfp42), Klf2, Klf4, Prdm14, Stat3, Essrb, and Nr0b1 are all expressed at lower levels in AX-ESCs, more similar to ESCs in serum/LIF medium (Figs. 2A, 2C and S3A), indicating that AX-ESCs have lost naïve stem cell gene signatures and likely exited from the naïve pluripotent state.

We performed RNA-seq of 2i-ESCs and AX-ESCs, finding 1,780 genes that were differentially expressed, and which are significantly enriched in pluripotency genes and the MAPK signaling pathway (Fig. S3B–D). Among these genes, 1,108 genes were upregulated in AX-ESCs, including most formative genes (Fig. 2D). Expression levels of formative genes such as Wnt8a, Pou3f1, Pim2, Sall2, Sox3, c-Myc, Eras, and Lef1, were all significantly higher in AX-ESCs, but silenced or expressed at low levels in 2i-ESCs (Figs. 2B, 2D, and S3B). Notably, genes expressed exclusively in primed stem cells, were not expressed in either 2i-ESCs or AX-ESCs (Fig. S3E).

Expression levels for Dnmt3a, Dnmt3b and Dnmt3l are all higher in AX-ESCs compared to ESCs cultured otherwise, both in transcripts (Fig. S3B and S3F) and protein (Fig. 2E). In agreement with these results, compared to the reduced total 5-methylcytosine (5mC) in 2iL ESCs, AX-ESCs exhibit a significant increase similar to levels seen with serum/LIF ESCs (Fig. 2F). Reduced-representation bisulfite sequencing (RRBS-Seq) confirmed the increase of global DNA methylation at promoters (Fig. 2G) and CpGs (Fig. 2H) in AX-ESCs. Of 5,951 differentially methylated promoters, 5937 were hyper-methylated in AX-ESCs (>99%).

Responsiveness to inductive cues for lineage specification is another prominent feature that distinguishes naïve and formative stem cells (Smith, 2017). To measure responsiveness, we adopted a standard directed differentiation protocol. ESCs were cultured in serum-free differentiation (SFD) medium to form embryoid bodies (EBs), and Activin A was added at either day 0, day 1 or day 2 to induce differentiation toward mesendoderm, identified by Brachyury (T) expression (Fig. 2I). T positive cells were analyzed by flow cytometry to determine the differentiation efficiency. As expected, 2i-ESCs were not responsive to stimulus in the Day 0 group, failing to generate T positive cells (<0.1%) at the 4 timepoints (12 h, 24 h, 36 h, 48 h post Activin A induction) (Fig. 2J). Intriguingly, AX-ESCs were responsive to Activin A induction though not at high efficiency; approximately 5% T-positive cells were found at 48 h post induction (Fig. 2J). Notably, differentiation efficiency was remarkably increased for AX-ESCs when induced at Day 1 (after 24 h in SFD medium), generating over 40% T-positive cells at 36–48 h post induction (Fig. 2J), whereas induction of the 2i-ESCs remained inefficient (~5%) (Fig. 2J). The differentiation efficiency for 2i-ESCs significantly increased and reached comparable levels of AX-ESCs only when induced at Day 2 (Fig. 2J), both showing similar differentiation kinetics and producing over 20% T-positive cells 42 h after induction (Fig. 2J). These results are consistent with AX-ESCs acquiring a formative-like pluripotent state that ensures relatively rapid response to inductive cues and execution of lineage fate decisions. Taken together, the results indicate that AX-ESCs lose naïve stem cell identity and acquire a new formative-like intermediate state of pluripotency.

### Lipids in AX drive pluripotency transition

**Figure 3.**
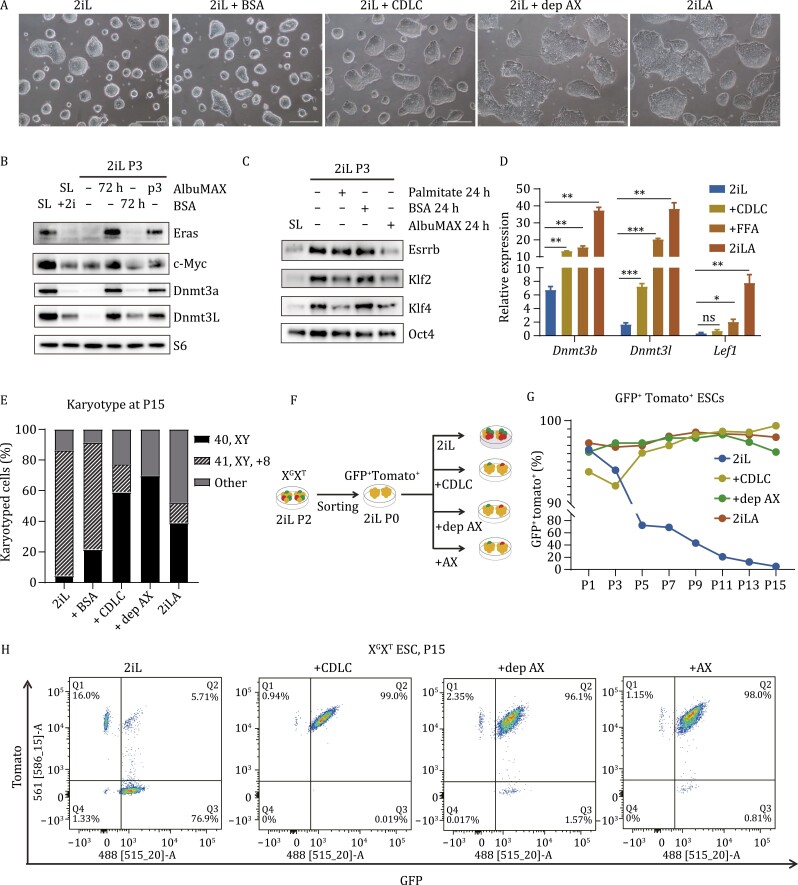
**Lipids in AX drive pluripotency transition and promote genomic stability.** (A) Colony morphology is shown for ESCs cultured in 2iL, 2iL + BSA (0.1% fatty acid free BSA), 2Il + CDLC (0.1%BSA + 2% chemical defined lipid component), 2iL + dep-AX (deproteinized AX), and 2iLA. Scale bar, 25 µm. (B) Western blot assay to evaluate expression levels of Eras, c-Myc, Dnmt3a and Dnmt31. (C) Western blot assay to evaluate expression levels of Esrrb, Klf2, Kif 4 and Oct4. (D) qRT-PCR expression levels of the formative marker genes *Dnmt3b*, *Dnmt3l*, and *Lef1* in ESCs cultured in 2iL, 2iL + CDLC, 2iL + dep-AX, or 2iLA. (E) Karyotyping analyses of ESCs at P12 after culture in 2iL, 2iL + BSA (0.1%fatty acid free BSA), 2iL + CDLC (0.1% BSA +2% chemical defined lipid component), 2iL + dep-AX (deproteinized AX), or 2iLA. (F) Schematic of the experiments using X^G^X^T^ reporter ESCs to monitor the X chromosome loss. (G) FACS analyses of X chromosome loss for X^G^X^T^ ESCs cultured in different media. (H) Flow cytometry analysis of the X^G^X^T^ ESCs showing GFP + Tomato + ESCs at passage 15.

Previous studies of human pluripotent stem cells demonstrated that lipids in AX, not albumin (BSA), support self-renewal and facilitate pluripotent cell transitions (Garcia-Gonzalo and Belmonte, 2008; Cornacchia et al., 2019). To test whether lipids are essential in the murine system, we first deproteinized AX (dep-AX) in a manner that can efficiently remove proteins including albumin from AX, as previously described (Cornacchia et al., 2019). ESCs cultured in 2iL medium supplemented with dep-AX retained a formative colony morphology (Fig. 3A), while ESCs cultured in 2iL supplemented with fatty acid free BSA (BSA) maintained a naïve colony morphology (Fig. 3A). Addition of chemical defined lipid components (CDLC) plus BSA in 2iL medium, specifically Lipid Mixture 1 which contains non-animal derived fatty acids (arachidonic, linoleic, linolenic, myristic, oleic, palmitic, stearic, cholesterol, tocopherol acetate and Pluronic) could also induce the change of colony morphology (although not as dramatic as with AX), clearly distinct from naïve colony morphology, being flatter and much more irregularly shaped (Fig. 3A). Therefore, lipids, not albumin, induce the change of colony morphology for murine ESCs cultured in 2iLA medium.

We next analyzed the impact of lipid supplementation on expression of pluripotency genes. Western blot analysis showed that the protein levels of formative genes Eras, c-Myc, Dnmt3a, and Dnmt3L were increased (Fig. 3B) while those of naïve genes Esrrb, Klf2, and Klf4 were decreased in 2iL medium supplemented with AX or the saturated fatty acid palmitate (Fig. 3C); supplementing only BSA in 2iL medium had no effect on expression of these genes (Fig. 3B and 3C). qPCR assays demonstrated that levels of the formative genes *Dnmt3s* and *Lef1* were enhanced in 2iL medium supplemented with CDLC, free fatty acids (FFA) or AX (Figs. 3D and S3F). Therefore, lipids induce the expression of formative genes and decrease expression of naïve genes, indicating that lipids are sufficient, independent of BSA, to induce the pluripotency transition in 2iLA medium.

In addition to increased steady-state levels of nucleotide pools, LC–MS analysis also showed that treatment with AX enhanced the steady-state levels of the non-canonical TCA cycle metabolites citrate and cis-aconitic acid (Fig. S4A and S4B), which were recently reported to be engaged in the exit of naïve pluripotency (Arnold et al., 2022). qRT-PCR analysis of TCA cycle enzymes showed that AX treatment did not alter expression of most genes with the exception of succinate dehydrogenase (*SDHB*), which was upregulated after just 24-h treatment and sustained through passage 3 (Fig. S4C). In serum-free 2iL medium cells must expend high levels of reducing equivalents in the form of NADPH to synthesize lipids de novo. Consistent with high levels of de novo lipogenesis, ACLY, FASN, SCD1, SCD2 protein levels are increased in 2iL while treatment with AX induced a strong downregulation indicating a reduction in capacity for lipid biosynthetic reactions (Fig. S4D and S4E). Moreover, two metabolic intermediates associated with lipid metabolism, glycerol-3-phosphate and phosphoethanolamine, were also reduced in AX treated cells (Fig. S4F). LC–MS also revealed a steady-state reduction in amino acid abundance (Fig. S4G), potentially a result of increased anabolic reactions and protein synthesis in AX-ESCs expressing high levels of c-Myc and ERas.

### Lipids in AX promote genome stability and prevent X chromosome loss in female ESCs

We next evaluated the effect of lipids on genome stability. Male ESCs expanded under 2iL (un-supplemented), AX, dep-AX or CDLC conditions were karyotyped at P12. As expected, the XY ESCs cultured in 2iL medium became aneuploid (95%), while 40% of cells in 2iLA medium maintained a normal karyotype (Fig. 3E). Medium supplemented with CDLC or dep-AX promoted retention of normal ESC karyotype (60% and 70%, respectively) (Fig. 3E). These results demonstrated that both CDLC and dep-AX can significantly promote the karyotypic stability of male ESCs comparable to AX supplementation of 2iL medium.

To investigate the effect of lipids on X chromosome loss in female ESCs, we adopted the X^G^X^T^ double reporter system to monitor X chromosome loss during *in vitro* culture (Fig. 1F). The GFP and Tomato double positive cells were sorted at passage 2 in 2iL and expanded in 2iL for 2 passages. The cells were then split at the same density into 2iL medium alone, or supplemented with AX, dep-AX or CDLC (denoted as passage 0, P0), followed by FACS analyses to assess X chromosome loss over the course of *in vitro* expansion (Fig. 3F). The double positive cell population was incrementally depleted from P1 to P15 in 2iL medium, but it was maintained at a high percentage (over 95%) in 2iLA medium (Fig. 3G). Notably, both dep-AX and CDLC supplementation also preserved a high double positive cell population (over 90% at P15) (Fig. 3H), showing that lipids efficiently prevent X chromosome loss in supplemented 2iL medium. Therefore, lipids can mitigate aneuploidy and stabilize the genome of murine ESCs cultured in 2iL medium, supporting the notion that lipids are responsible for the effects of AX on genomic stability.

### Erk2 phosphorylation is essential for lipid-induced pluripotency transition

To determine whether lipids or lipid metabolism (lipid metabolites) drive pluripotency transition, we inhibited carnitine palmitoyltransferase (CPT1) with Etomoxir (ETO) (Fig. S5A), an irreversible inhibitor of CPT1 on the inner face of the outer mitochondrial membrane (Kruszynska and Sherratt, 1987). Pluripotency transition from naïve to formative-like state was observed in ESCs cultured in 2iLA medium supplemented with ETO (Fig. S5B and S5C). ACAA2 catalyzes the last step of the mitochondrial fatty acid beta oxidation spiral (Fig. S5A); inhibition of ACCA2 with Trimetazidine (TMZ) (Kantor et al., 2000) also did not block the pluripotency transition in 2iLA medium (Fig. S5B). To validate these results, we took a genetic approach by disruption in ESCs of the *Cpt1a* gene using CRISPR/Cas9 (Fig. S5D). Consistent with inhibitor results, the *Cpt1a* null ESCs displayed a formative-like colony morphology in 2iLA medium (Fig. S5E) and increased expression levels of formative gene *Dnmt3b*, based on qRT-PCR (Fig. S5F). Therefore, fatty acid oxidation is dispensable for the lipid-induced pluripotency transition.

**Figure 4.**
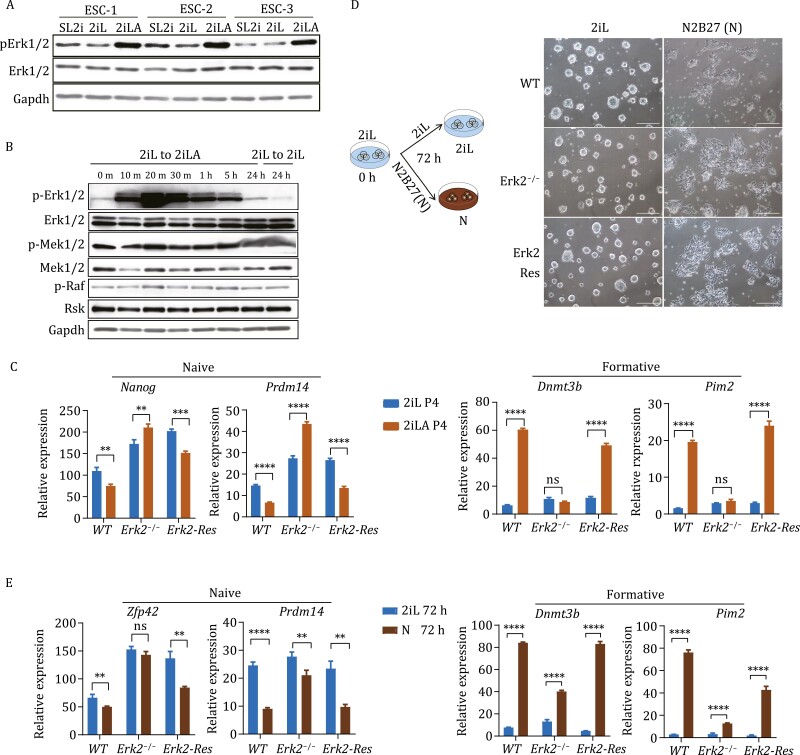
**Erk2 phosphorylation is essential for lipid-induced pluripotency transition.** (A and B) Western blot for Erk1/2 and p-Erk1/2 proteins in ESCs cultured in 2iL or 2iLA at passage 3 (A) or for Mek/Erk signaling related proteins in ESCs after switching 2iL to 2iLA (B). m, min; h: hours. (C) qRT-PCR for the naive marker genes *Nanog*, *Prdm14* and formative marker genes *Dnmt3b*, *Pim2* for *WT*, *Erk2*^−/−^, *Erk2-Res* ESCs at passage 4 cultured in 2iL or 2iLA. *Erk2-Res*: *Erk2*-rescued in the *Erk1*/*2* mutant ESCs (Lentiviral-based shRNA against *Erk1* in the *Erk2*^−/−^ ESCs). Experiments were repeated three times (*n* = 3), ns: no significant difference, ^*^P < 0.05, ^**^P < 0.01, ^***^P < 0.001, ^****^P< 0.0001, two-tailed *t*-test was used. (D and E) Schematic illustration of the experimental design and ESC colony morphology change (D), qRT-PCR for the naive marker genes *Zfp42*, *Prdm14*, and the formative marker genes *Dnmt3b*, *Pim2* for WT, and *Erk2*^−/−^*Erk2-Res* (E) after 2i and LIF removal for 72 h. *n* = 3 experiments.

While Mek/Erk signaling is thought to be inhibited by PD in 2iLA medium, Western blot assays indicated that phosphorylated-Erk1/2 (p-Erk1/2) was significantly increased in ESCs cultured in 2iLA medium compared to serum/LIF+2i medium (Fig. 4A). This suggests that AX blunts the effect of PD on the inhibition of MAPK signaling. The kinetics of AX and p-Erk1/2 signaling was explored by performing a time course experiment focused on genes that are upstream of Erk1/2. Mek1/2, p-Mek1/2, and p-Raf (Erk upstream genes), did not respond to AX addition at the protein level, while levels of p-Erk1/2 increased as early as 10 min and peaked from 20 min through 5 h post AX supplementation (Fig. 4B), with higher p-Erk1/2 levels maintained in 2iLA medium thereafter (Fig. 4B). Notably, qPCR analyses showed that expression of *Dnmt3a*/*b*/*l* were not changed until 1 h after AX addition (Fig. S6A).

To investigate if Erk1/2 signaling is involved in the pluripotency transition induced by lipids, we used an ESC line in which *Erk2* is constitutively deleted and *Erk1* is knocked down by shRNA (designated *Erk1*/*2* mutant) (Tee et al., 2014). With the *Erk1*/*2* mutant, both Erk1 and Erk2 proteins are depleted, while Erk2 activity can be rescued by adding back the *Erk2* cDNA (Erk2-Rescue, *Erk2-Res*) (Fig. S6B), as confirmed by the expression of *Spry4*, a direct target of the Erk1/2 signaling pathway (Fig. S6C). When culturing *Erk1*/*2* mutant ESCs in 2iLA medium, the expression levels for formative genes *Wnt8a and Lef1* were relatively unchanged compared to the 2iL controls (Fig. S6D) and the *Erk2*^−/−^ ESCs still maintained relatively dome-like morphology (Fig. S6E), suggesting that pluripotency transition is likely delayed in 2iLA medium in these cells.

Erk1/2 signaling is essential for self-renewal and maintenance of ESCs, and double knockout of *Erk1* and *Erk2* is not tolerated *in vitro* (Chen et al., 2015). To circumvent this problem, we analyzed single *Erk2* knockout, as *Erk2* is thought to be the key gene regulating pluripotency transition (Kunath et al., 2007; Hamilton and Brickman, 2014). Gene expression analysis showed that *Erk2* deletion is sufficient to block response to lipid-induced pluripotency transition (Fig. S6D). Remarkably, the pluripotency transition was severely impaired in *Erk2*^−/−^ ESCs induced by lipids even at passage 4, as indicated by the sustained expression of naïve genes such as *Nanog* and *Prdm14*, while the expression levels of formative genes *Dnmt3b* and *Pim2* remained largely unchanged (Fig. 4C). To test if this defect is specific to lipid-induced transition, we induced the *Erk2*^−/−^ ESC differentiation by removal of 2i and LIF from the basic N2B27 culture medium (Fig. 4D). Under these conditions, *Erk2*^−/−^ cells initiated differentiation and formed differentiated colonies similar to WT ESCs at 72 h (Fig. 4D). qRT-PCR analyses of the *Erk2*^−/−^ cells showed that the expression levels of naïve genes *Prdm14* and *Zfp42* were downregulated while those for the formative genes *Dnmt3b* and *Pim2* were significantly upregulated (Fig. 4E). However, the transcriptional changes in *Erk2*^−/−^ cells after switching to N2B27 medium were relatively modest compared to WT cells, suggesting a delay in the pluripotency transition, as previously described for multiple mutants in the Fgf/Erk pathway including *Erk1* and *Erk2* single mutants (Leeb et al., 2014). Our results indicate that lipids stimulate Erk2 phosphorylation, and this is essential for lipid-induced pluripotency transition.

### Male and female all-ESC mice are generated from de novo derived ESCs using AX-based medium

**Figure 5.**
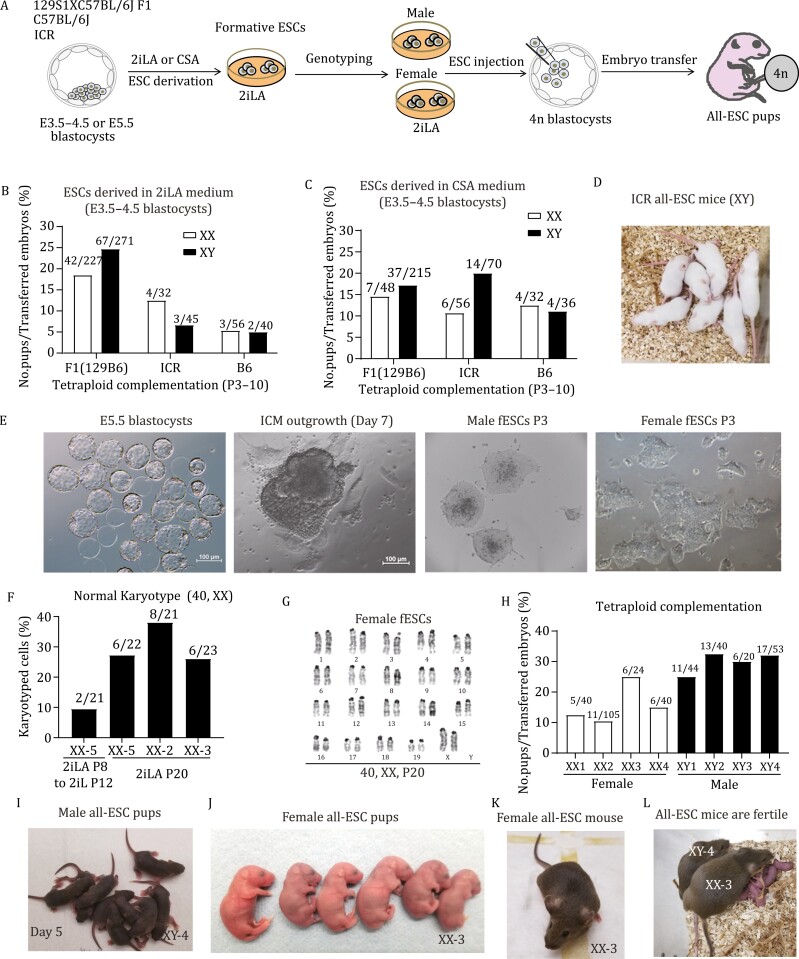
**Generation of male and female all-ESC mice from *de novo* derived ESCs using AX-based medium.** (A) Schematic illustration of the experimental design. (B) Tetraploid complementation assays for de novo derived ESCs (E3.5–5.5 embryos) in AX-based medium. F1 (129B6): hybrid F1 crossed with C57B6 females to 129S1 male; ICR: outbred strain; B6: inbred strain C57BL/6j; XX: female; XY: male. The numbers for each bar represent the No. pups/No. embryos transferred. (C) Tetraploid complementation assays for *de novo* derived ESCs (E3.5–4.5 embryos) in CSA medium. (D) A litter of all-ESC mice from a male ESC line derived from E4.5 ICR blastocysts in CSA medium. (E) E5.5 blastocysts cultured *in vitro* (embryos hatching from the zona pellucida). The ICM outgrowth on feeders in 2iLA (second panel). The colonies of male fESCs (formative ESCs, third panel) or female fESCs (fourth panel) derived from E5.5 blastocyst are shown in 2iLA at passage 3. Scale:100 μm. (F and G) Karyotyping analyses of the embryo-derived female ESCs at P20. (H) Tetraploid complementation assays for embryo-derived male and female ESCs. The numbers for each bar represent the No. pups/ No. embryos transferred. (I) A litter of pups from XY-4 male cell line is shown at day 5 that was naturally delivered at term (9 pups/15 embryos transferred). (J) A litter of embryo-derived female fESCs (XX-3) is shown at day 1 (6 pups/24 embryos transferred). (K) Adult female from the embryo-derived ESCs (XX-3). (L) Both male and female mice generated from the embryo-derived ESCs are fertile and produce normal litters. Bar scale in panel E: 100 μm.

We next explored derivation of ESCs from E3.5–4.5 embryos from different strains using 2iLA and assessed the developmental potential by tetraploid complementation (Fig. 5A). After removal of the zona pellucida, E3.5–4.5 embryos were placed into a 96-well plate on feeders and cells expanded in 2iLA medium (Fig. S7A–D). Male and female ESC lines were derived from F1 (129X B6) using 2iLA medium. All-ESC mice can be generated from both male and female cell lines to obtain live pups, with 24.7% and 18.5% efficiency, respectively (live pups/embryos transferred, Figs. 5B and S7E). Both male and female all-ESC pups appeared normal and survived to adulthood. We also derived ESCs from the inbred C57B6 strain using 2iLA medium and found all-ESC pups can be generated from these, although not as efficiently as with the F1 hybrid strain (Fig. 5B). The outbred ICR line has been considered a non-permissive strain to derive ESCs (Lee et al., 2012; Han et al., 2020). Using 2iLA medium, we efficiently derived ESC lines from E3.5–4.5 ICR blastocysts. Both male and female ICR ESC lines generated all-ESC pups via tetraploid complementation with an efficiency of 6.7% for male and 12.5% for female (Figs. 5B and S7F). Our studies demonstrated that supplementing AX into 2iL medium (2iLA) enabled derivation of both male and female ESC lines with full potential to generate all-ESC pups, suggesting that lipids maintain genomic stability and developmental potential during ESC derivation.

To test if PD can be replaced by Erk1/2 inhibitors for ESC derivation, we substituted PD with the p-Erk1/2 inhibitor SCH772984 in 2iLA medium (CHIR/SCH772984/LIF/AlbuMAX, designated CSA). ESC lines were readily generated from F1, ICR, and B6 strains with an efficiency as high as with 2iLA medium (Fig. S7C and S7D). The efficiency to obtain all-ESC pups were improved for the non-permissive strains of ICR (XX: 10.7%, XY: 20.0%) and B6 (XX: 9.4%, XY: 11.1%), while a similar efficiency as in 2iLA medium was achieved for the F1 strain (Figs. 5C, 5D and S7F). All-ESC mice generated from both male and female ESCs (F1, ICR, and B6) were normal and fertile (Fig. 5C and 5D). Our results demonstrate that PD, the Mek1/2 inhibitor, can be substituted with Erk1/2 inhibitors for ESC derivation, suggesting that p-Erk1/2 direct inhibition and Wnt agonist are sufficient to preserve pluripotency for ESC derivation and maintenance in AX medium.

Finally, we derived ESC lines directly from late-stage embryos (E5.5) using 2iLA medium and tested the cell lines for 4n competence through generation of all-ESC mice (Fig. 5A). We obtained and cultured 23 hatching blastocysts (E5.5) in the stage of embryonic escape from the zona pellucida (Fig. 5E). Embryos were placed individually into a 96-well plate on feeders and cells expanded in 2iLA medium. The ICM outgrowths emerged from all embryos at day 7 and cell lines were successfully established from each embryo (Fig. 5E). The colony morphology of these cell lines was distinct from ESCs cultured in 2iL medium and resembled the formative morphology of ESCs cultured in 2iLA medium (Fig. 5E). Notably, colonies from female cell lines were flatter and more irregular-shaped than those from male cell lines (Fig. 5E). The female cell lines derived in 2iLA medium were karyotyped and they maintained 25%–35% normal 40, XX karyotypes at passage 20, while nearly all cells (over 90%) became aneuploid following 12 passage culture in 2iL medium, even if they were first cultured in 2iLA for 8 passages (Fig. 5F and 5G). Therefore, both male and female ESC lines can be efficiently derived from E5.5 blastocysts with improved genomic stability when maintained in 2iLA medium.

We evaluated the developmental potential of male and female ESC lines derived from late-stage blastocysts. All 4 male lines tested generated all-ESC pups with efficiencies from 25% to 30% (Fig. 5H). The pregnant surrogates carrying these embryos delivered naturally at term and the all-ESC pups were healthy and grew normally to fertile adults (Fig. 5I). Female ESC lines generated all-ESC pups through tetraploid complementation with efficiencies from 10% to 25% (Fig. 5H). Female all-ESC pups were normal and survived to adulthood (Fig. 5J and 5K), producing normal litters when crossed with male all-ESC mice (Fig. 5L). These results affirm that full pluripotency and 4n competence can be preserved in both male and female ESCs derived from late-stage blastocysts in 2iLA medium.

## Discussion

Stabilization of pluripotency for both male and female ESCs during long-term culture would benefit research into the mechanisms of cell fate determination, epigenetic reprogramming, and modeling of early development using synthetic embryos. We show that supplementing 2i/LIF medium with lipid-rich albumin AlbuMAX significantly improves the genomic stability and developmental potential of murine ESCs. ESCs cultured in 2i/LIF + AX (2iLA) medium can be propagated in a state highly similar to formative pluripotency. Mechanistically, lipids directly stimulate Erk2 phosphorylation, leading to exit of naïve state and establishing formative-like pluripotency for ESCs cultured in 2iLA medium. Lipid metabolism through β-oxidation is dispensable for transition from naïve to formative-like state. Lipid metabolism reduces the lipogenesis and amino acid biosynthesis and promotes non-canonical TCA metabolites recently reported to be involved in pluripotency transition (Arnold et al., 2022). Lipid metabolism promotes nucleotide and Acyl-CoA biosynthesis, enhances the expression of DNMT3s that are involved in the maintenance of telomere length and DNA methylation, thereby improving genome stability during long-term culture. Stimulated Erk2 activity by lipids also alleviates X chromosome loss and possibly trisomy for ESCs cultured in 2iLA medium. The dual role of lipids on genome stability and pluripotency facilitates the preservation of 4n competency of murine ESCs for both sexes during long-term culture *in vitro* (Fig. S8). Successful generation of healthy, fertile female all-ESC mice from *de novo* derived ESCs demonstrates that AX-based culture media support derivation of fully potent murine ESCs of both sexes. Interestingly, in this AX-based system, PD, the Mek inhibitor, can be substituted with an Erk inhibitor (CSA), for efficient ESC derivation from various mouse strains.

As previous studies showed, long-term propagation of mouse ESCs in 2i/LIF medium leads to increased genome instability such as an increase of aneuploidy (mainly trisomy and X chromosome loss) and loss of imprinting, as well as global DNA hypomethylation (Choi et al., 2017; Yagi et al., 2017). Here we identified a biosynthetic pathway such as endogenous nucleotide pool depletion in 2i/LIF cultures that might also contribute to genome instability in 2i-ESCs. This conclusion is supported by the observation of extensive autosomal chromosome loss (both pairs for chromosomes 2, 4, 10, 15, 16, 17, 19) in 2i-ESCs at P43 (Figs. 1E and S2E), this was significantly improved in AX-ESCs that exhibit increased nucleotide pools (Figs. 1H and S2E). Nucleotide pool depletion has been well documented to contribute to genome instability (Magdalou et al., 2014; Fasullo and Endres, 2015; Pai and Kearsey, 2017; Halliwell et al., 2020). In this study, when we added exogenous nucleoside into 2i/LIF medium, we observed improvement of colony morphology over passaging, but not a rescue of trisomy aneuploidy based on karyotyping at P15 (Fig. S2H). However, it is difficult to measure the effects of nucleotide addition to genome stability at the molecular level. For example, nucleotide pool depletion can lead to DNA replication and repair stress, and telomere shortening as well (Gupta et al., 2013; Brind’Amour et al., 2015; Pai and Kearsey, 2017; Maciejowski and de Lange, 2019), which are collectively contributed to the genome instability. Moreover, nucleotide pools are normally tightly regulated in cells with salvage pathways for nucleotide deficiency, in addition to nucleotide recycling pathways. It is expected that cells cultured in 2i/LIF would not immediately cause chromosome loss but instead result from cumulative mutations over extended passaging, which could partially explain why extensive autosomal chromosome loss was only observed in late passages in 2i-ESCs (>P25). Therefore, our study provided novel evidence that nucleotide pool depletion is associated with genome instability of ESCs cultured in 2i/LIF, in addition to previously reported loss of imprinting and hypomethylation. AX addition improves genome stability, and this is associated with an increased nucleotide pool.

It is known that lipids serve as signaling molecules in regulation of the Ras-Raf-Mek-Erk pathway (Anderson, 2006; Garcia-Gonzalo and Belmonte, 2008). Our data show that Erk1/2 rapidly senses the addition of AX to elevate p-Erk1/2 independently of upstream Raf and Mek (Fig. 4B), and Mek1/2 is essential for the phosphorylation of Erk1/2, indicating that lipids regulating Erk2 phosphorylation might act through changing Mek1/2 kinase domain conformation, which is thought to be inhibited by PD. However, whether this is an effect induced by specific types of lipids needs to be further studied.

The discovery of the 2i system (Ying et al., 2008) revolutionized both stem cell-culture and derivation, not only for mice but also other mammalian species including humans (Buehr et al., 2008; Li et al., 2008; Czechanski et al., 2014; Ware et al., 2014; Van der Jeught et al., 2015; Guo et al., 2016). Yet the 2i system has also been found to cause irreversible genetic and epigenetic changes in murine ESCs (Choi et al., 2017; Yagi et al., 2017). The modified 2i systems (2iLA and CSA), supplemented with lipid-rich albumin, can efficiently prevent the detrimental effects of 2i and preserve pluripotency while maintaining genomic stability. This improvement provides a reliable culture system for targeting of ESCs and generating genetically modified mice, and also for modeling developmental processes, which depend on the genetic and phenotypic fidelity of ESCs during propagation. Female cells are typically sensitive to 2i resulting in loss of an X chromosome and DNA methylation; thus, female ESC lines derived using 2i have lost developmental potential (Choi et al., 2017; Yagi et al., 2017). The modified 2iLA system can efficiently derive female cell lines that retain the potential to generate fertile all-ESC mice, suggesting that the genetic and epigenetic fidelity has been preserved. Therefore, the 2iLA system can be used for de novo derivation of pluripotent stem cell lines including even for non-permissive strains.

In summary, our findings underscore the importance of lipids in cell-culture media for maintenance of genomic, epigenomic and phenotypic integrity, and discover novel mechanisms governing pluripotent cell transitions that are conserved in early mammalian development from mice to humans.

## Supplementary Material

pwad008_suppl_Supplementary_Material

pwad008_suppl_Supplementary_Tables

## Data Availability

All the RNA-seq and RRBS data have been uploaded to the NBCI BioProject. The accession number is uploaded to the Gene Expression Omnibus website. The accession number is PRJNA806916.

## Abbreviations

2iL: 2i/LIF
2iLA: 2i/LIF/AlbuMax
2i-ESCs: ESCs cultured in 2i/LIF medium
AX: AlbuMAX
AX-ESCs: ESCs cultured in 2i/LIF/AlbuMAX medium
BSA: bovine serum albumin
CDLC: chemically defined lipid concentrate
CSA: CHIR/SCH772984/LIF/AlbuMAX
dep-AX: deproteinized AlbuMAX
ESCs: embryonic stem cells
ETO: Etomoxir
fESCs: formative embryonic stem cells
ffBSA: fatty acid free BSA
fPSCs: formative pluripotent stem cells
LC–MS: liquid chromatography–mass spectrometry
LIF: leukemia inhibitory factor
N: N2B27 media
P: passage
RRBS: reduced-representation bisulfite sequencing
SFD: serum-free differentiation media
SL: serum/LIF
SL2i: serum/LIF/2i
WB: Western blots
X^G^X^T, ^: X^GFP^X^Tomato^ ESC
