# Supplementary material for: Dual role of lipids for genome stability and pluripotency facilitates full potency of mouse embryonic stem cells
Source: Protein Cell. 2023 Feb 16;14(8):591–602. doi: 10.1093/procel/pwad008 (PMC10392030; doi:10.1093/procel/pwad008)
Supplement: pwad008_suppl_Supplementary_Material [file pwad008_suppl_supplementary_material.pdf]

Supplementary Materials for

Dual Role of Lipids for Genome Stability and Pluripotency Facilitates Full Potency of Mouse Embryonic Stem Cells

Liangwen Zhong[1], Miriam Gordillo[2, ‡], Xingyi Wang[4, ‡], Yiren Qin[1, ‡], Yuanyuan Huang[1, ‡], Alexey Soshnev[3,6], Ritu Kumar[2,7], Gouri Nanjangud[5], Daylon James[1], C. David Allis[3], Todd Evans[2, *], Bryce Carey[3, *], Duancheng Wen[1, *]

This PDF file includes:

Methods, Fig. S1-S8, References

Methods

Mice and embryos. Animals were housed and cared for according to a protocol approved by the IACUC of Weill Cornell Medical College (Protocol number: 2014-0061). Wild-type ICR mice were purchased from Taconic Farms (Germantown, NY); the 129S1 and C57B6 mice were purchased from Jackson Laboratories. Females were super-ovulated at 6–8 weeks with 0.1 ml CARD HyperOva (Cosmo Bio Co., Cat. No. KYD-010-EX) and 5 IU hCG (Human chorionic gonadotrophin, Sigma-Aldrich) at intervals of 48 hours. The females were mated individually to males and checked for the presence of a vaginal plug the following morning. Plugged females were sacrificed at 1.5 days post hCG injection to collect 2-cell embryos. Embryos were flushed from the oviducts with advanced KSOM (Cat # MR-101-D, Millipore) and cultured in KSOM medium in an incubator under 5% CO_2 at 37°C until blastocyst stage for ESC injection.

Blastocyst injection and tetraploid complementation. ESCs were trypsinized, resuspended in ESC medium and kept on ice. A flat tip microinjection pipette was used for ESC injections. ESCs were collected at the end of the injection pipette and 10–15 cells were injected into each blastocyst. The injected blastocysts were kept in KSOM until embryo transfer. Typically, ten injected blastocysts were transferred into each uterine horn of 2.5 dpc pseudo-pregnant ICR females. Tetraploid embryos were generated using 2-cell stage embryos flushed from the oviducts. The 2-cell embryos were subjected to electrofusion to induce tetraploidy. Fused embryos were moved to new KSOM micro drops covered with mineral oil and cultured to blastocyst stage until ESC injection.

2iL medium and 2iLA medium. 2iL medium contains 1:1 mixture of DMEM/F12 and Neurobasal media supplemented with N2 (Gibco™, 17502048) and B27 (Gibco™, 17504044), L-glutamine (Millipore, TMS-002-C), 2-mercaptoethanol (Millipore, ES-007-E), 1x penicillin/streptomycin (Millipore, TMS-AB-2C) and LIF (Sigma-Aldrich, ESG1107), 1 μM PD0325901 (Sigma or Stemolecule) and 3 μM CHIR99021 (Sigma). 2iLA medium was made from 2iL medium supplemented with 1% AlbuMax (W/V, Gibco™, 11020039).

ESC derivation and culture. The original ESC lines used in this study were derived from hybrid F1 fertilized embryos by crossing C57BL/6J females and 129S1 males. The ESC derivation medium contains KnockOut™ Dulbecco's modified Eagle's medium (Gibco, 10829-018), 20% KnockOut™ Serum Replacement (Gibco, 10828), 10^3 IU recombinant mouse LIF (Millipore, ESG1107), 2mM L-glutamine (Millipore, TMS-002-C), 1x penicillin/streptomycin (Millipore, TMS-AB-2C), 1x non-essential amino acids (Millipore, TMS-001-C), 1x nucleosides for ES cells (Millipore, ES-008-D), 1x β-mercaptoethanol (Millipore, ES-007-E), 1μM PD98059 (Promega, V1191) and 3 μM CHIR99021 (Sigma). Briefly, E3.5-E5.5 blastocysts were collected from the plugged females treated with CARDova/hCG. The zona pellucida was removed by briefly exposing the blastocysts to Tyrode's acidic solution (Millipore, MR-004). Blastocysts with dissolved zona pellucida were picked up with a mouth pipette and washed in KSOM medium for 2-3 times. Blastocysts were then placed individually on 96-well plate on feeder layers with the derivation medium. After 5-7 days' culture, the ICM outgrowth originated from the blastocysts were trypsinized in 20 μL 0.025% trypsin solution (Millipore, SM-2004-C) for 5 min, and 200 μL culture medium was added to stop the

reaction. Colony expansion of ESCs proceeded from 48-well plates to 6-well plates with feeder cells. Cell aliquots were cryopreserved using Cell Culture Freezing Medium (Millipore, ES-002-D) and stored in liquid nitrogen. ESCs were routinely expanded and maintained as following: Before passaging, the plates were treated with EmbryoMax™ 0.1% gelatin solution (Cat # ES-006-B, Millipore) for at least 10 min, and the ESC culture media were added to the wells after removal of gelatin solution. ESCs were then placed on the wells with proper cell density and usually cultured for 2 days before next passaging.

Mutant ESC lines. The $X^{GFP}X^{Tomato}$ ESC line was a gift from Dr. Konrad Hochedlinger and the *Erk1/2* mutant ESC lines were obtained from Dr. Danny Reinberg with the permission from Dr. Sylvain Meloche. The Nanog-GFP cell line was a gift from Dr. Rudolf Jaenisch. The *Cpt1a*$^{-/-}$ ESC cell line was generated by CRISPR-Cas9 using Neon transfection system, the sgRNA sequencing is CACCACGATAAGCCAGCTGGAGG.

Deproteinization of AlbuMAX. AlbuMAX deproteinization was performed as described in Garcia-Gonzalo and Izpisu´ a Belmonte (Garcia-Gonzalo and Belmonte, 2008). Briefly, 0.5 mL of a 100X solution of Trypsin from bovine pancreas (Sigma) was added to 50 mL of a 10% w/v solution of AlbuMAX in base medium (AX-Trypsin solution) without supplement and incubated for 30 minutes at 37℃ in a water bath. Then, 0.5ml of a 100X solution of Soybean Trypsin inhibitor was added to the AX-Trypsin solution and incubated at room temperature for 30 minutes. The deproteinized AlbuMAX solution was sterile filtered and added to 2iL to a final concentration of 1% w/v. Fatty acid-free BSA (Sigma, A3803) was added to 2iL medium to a final concentration of 0.1% w/v.

Chemically defined lipid concentrate (CDLC, Thermo Fisher Scientific, 11905031) was added at a concentration 2% v/v into 2iL media.

Karyotype analysis. Briefly, cultures were treated with Colcemid at final concentration of 0.05 µg/m. Following 60-90 min incubation, cells were trypsinized according to standard procedures, washed twice in 1X PBS, incubated in 0.075M KCl for 10 minutes at 37°C and fixed in chilled methanol-acetic acid (3:1). The fixed cell suspension was then dropped onto slides, stained in 0.08 µg/ml DAPI in 2xSSC for 3 minutes and mounted in antifade solution (Vectashield, Vector Labs). The stained slides were scanned using a Nikon E800 epifluorescence microscope equipped with imaging and digital karyotyping system from Applied Spectral Imaging (Carlsbad, CA). For each sample a minimum of 20 metaphases were captured. All metaphases were fully karyotyped and analyzed for chromosomal instability. The experiments were performed at the MSKCC Molecular Cytogenetics Core Facility.

RNA-sequencing. Total RNA was prepared from cultured ESCs using the RNeasy Mini Kit (QIAGEN) following the manufacturer's instructions. The RNA concentration and integrity were measured using a NanoDrop 2000 (Thermo Fisher Scientific) and Agilent 2100 Bioanalyzer (Agilent), respectively. The integrity of RNA was indicated by the RNA integrity number (RIN). RNA samples with sufficient concentration and RIN greater than 8.0 were further prepared for cDNA library preparation using poly-A selection and unstranded library preparation using a Truseq library preparation kit (Illumina) according to manufacturer's instructions. DNA library was then sequenced using a NovaSeq 6000 - S1 Flow Cell, with pair-end reads, 2x50 cycles at the Weill Cornell Genomics Core Facility. Reads from RNA-seq were aligned to mouse genome version mm10 using

TopHat. Gene counts were obtained using featureCounts from sort bam files, and only unique-mapping reads were used. Genes without any expression counts in any sample were discarded. Differentially gene expression analysis was performed using DESeq2 (version 1.4.5) R, and then detects differentially expressed genes (DEG) between 2i-ESCs and AX-ESCs groups (FDR < 0.1).

RRBS-sequencing. Reduced Representation Bisulfite Sequencing is a modification of the original RRBS protocol (Gu et al., 2011) and the in-house developed ERRBS method (Akalin et al., 2012) for base-pair resolution methylation sequencing analysis based on the use of a restriction enzyme to enrich for CpG fragments. 150 ng of RNA-free genomic DNA per sample was used for RBBS sequencing at Weill Cornell Medicine Epigenomics Core. Briefly, Concentration of double stranded DNA (dsDNA) was determined using Qubit Fluorometer, Perkin Elmer Labchip GX or agarose gel electrophoresis to determine molecular weight. 100 million (M) read per sample on a single end read flow cell with 100 sequencing cycles (SR100) was used for differential methylation analysis. FASTQ files were generated by bcl2fastq (V2.17) and filtered for pass filter reads based on Illumina’s chastity filter. Sequencing adapters were trimmed by FLEXBAR (V2.4) (Dodt et al., 2012), genomic alignments using Bismark (V0.14.4) (Krueger and Andrews, 2011) and Bowtie2 (V2.2.5) (Langmead and Salzberg, 2012) to reference mm10, and per base CpG methylation metrics were calculated with a custom PERL script. CpGs at a minimum threshold coverage of 5 reads were used for downstream analysis. More detail procedure can be obtained from Weill Cornell Epigenomics Core (https://epicore.med.cornell.edu/services.php?option=seqoverview#seq).

Western blotting assays. Cells were lysed into 1× SDS loading buffer (50 mM Tris-HCl pH 6.8,5% β-mercaptoethanol, 2% SDS, 0.01% bromophenol blue, 10% glycerol) followed by sonication (Bioruptor, 2 × 30 s at high setting). Proteins were resolved on a 5–15% gradient Tris–glycine SDS–PAGE gel and semi-dry-transferred to nitrocellulose membranes. The following primary antibodies were used at the indicated dilutions: Dnmt3a (Abcam, ab2850), Dnmt3b (Abcam, ab2851), GAPDH (CST, 5174, 1:10,000); Erk1/2 (CST, 4695); p-Erk1/2 (CST, 9101); Nanog (CST, D2A3 XP®). Horseradish peroxidase (HRP)-conjugated secondary antibodies and the ECL prime western blotting system (GE Healthcare, RPN2232) were used for detection of primary antibodies. For the Erk1/2 &p-Erk1/2 or Mek1/2&p-Mek1/2, the equivalent samples were loaded on two parallel gels synchronously and then the gels were transferred to nitrocellulose membranes synchronously. Chemiluminescent signals were captured with a digital camera (Kindle Biosciences).

Quantitative real-time PCR. ESCs were homogenized in RNA lysis buffer (RLT buffer, Qiagen) with 1% β-mercaptoethanol. RNA was then prepared following the RNeasy protocol (QIAGEN). DNA was digested using DNase I (QIAGEN) during the RNA extraction processes. The RNA concentration was measured by NanoDrop (Thermo Fisher Scientific). RNA (200 ng) was used for cDNA conversion using qScript Super Mix (Quanta Biosciences). After cDNA dilution with double-distilled H_2O at a 1:10 ratio, the qPCR reaction was prepared by mixing the gene-specific primers and PowerUp SYBR Green Master Mix (Thermo Fisher Scientific;cat. no. A25778). qPCR was run using a QuantStudio 3 machine (Thermo Fisher Scientific).

Flow cytometry. The attached cells were washed with 1x PBS and dissociated with Trypsin-EDTA (0.025%) at room temperature for 5 min. The same volume of Defined Trypsin Inhibitor (Gibco, R007100) was added to the detached cell suspension, incubated at room temperature for 5 min, and then washed with 1xPBS and centrifuged for the cell pellet. Samples without fluorescent reporter were used as the negative control. Finally, the cell pellet was suspended in MACS (1x PBS with 2mM EDTA and 0.5% BSA) with DAPI and applied to a flow cytometer for analysis (FACSymphony™ A5 or Fortessa , BD) at the Weill Cornell Medicine Flow Cytometry Core Facility .

ESC directed differentiation. For mesendoderm differentiation, embryoid bodies (EBs) were formed in serum free differentiation media (SFD). SFD consisted of 75% IMDM (Gibco), 25% Ham's F12 (Corning cellgro), 0.5X N2 (Gibco), 0.5X B27 (Gibco), 0.05% BSA (Gibco), 0.5mM Ascorbic Acid (Sigma), 2mM Glutamine (Corning), 0.45 mM monothioglycerol (Sigma), 100U/mL Penicillin and 0.1 mg/ml Streptomycin (Corning). On the first day of differentiation, cells were dissociated with Accutase (Sigma) and plated at 40,000 cells/ml in Petri dishes. Activin A (R&D Systems or Peprotech) was added at designed timepoints: day0 (0h), day1(24h), and day2 (48h) at a final concentration of 75 ng/ml. For Brachyury staining, EBs were dissociated with Accutase, fixed with 2% paraformaldehyde (Electron Microscopy Sciences) and stained with anti-Brachyury PE-conjugated Antibody (R&D) for 2h according to manufacturer instructions. After two washes, cells were analyzed using an Attune Nxt flow cytometer (Life Sciences).

Liquid chromatography–mass spectrometry (LC-MS). For all metabolite analyses, cells were seeded in 2iL or 2iLA media in 6-well plates. Metabolites were extracted with

1 ml ice-cold 80% methanol supplemented with 20 mM deuterated 2-hydroxyglutarate (D-2-hydroxyglutaric- 2,3,3,4,4-d5 acid (d5-2HG)) as an internal standard. After overnight incubation at -80℃, lysates were harvested and centrifuged at 21,000g for 20 min to remove protein. Extracts were dried in an evaporator (GenevacEZ-2Elite) and resuspended by incubation at 30℃ for 2h in 50 ml of 40 mg ml^{-1} methoxyamine hydrochloride in pyridine. Metabolites were further derivatized by addition of 80 ml of MSTFA plus 1% TMCS (Thermo Scientific) and 70 ml ethyl acetate (Sigma) and incubated at 37℃ for 30 min. Samples were analyzed using an Agilent 7890A GC coupled to Agilent 5975C mass selective detector in the Metabolomics & Lipidomics facility at The Rockefeller University. The GC was operated in split less mode with constant helium gas flow at 1 ml/min. One microliter of derivatized metabolites was injected onto an HP-5MS column and the GC oven temperature ramped from 60℃ to 290℃ over 25 min. Peaks representing compounds of interest were extracted and integrated using MassHunter software (Agilent Technologies) and then normalized to both the internal standard (d5-2HG) peak area and the protein content of duplicate samples as determined by a BCA protein assay (Thermo Scientific).

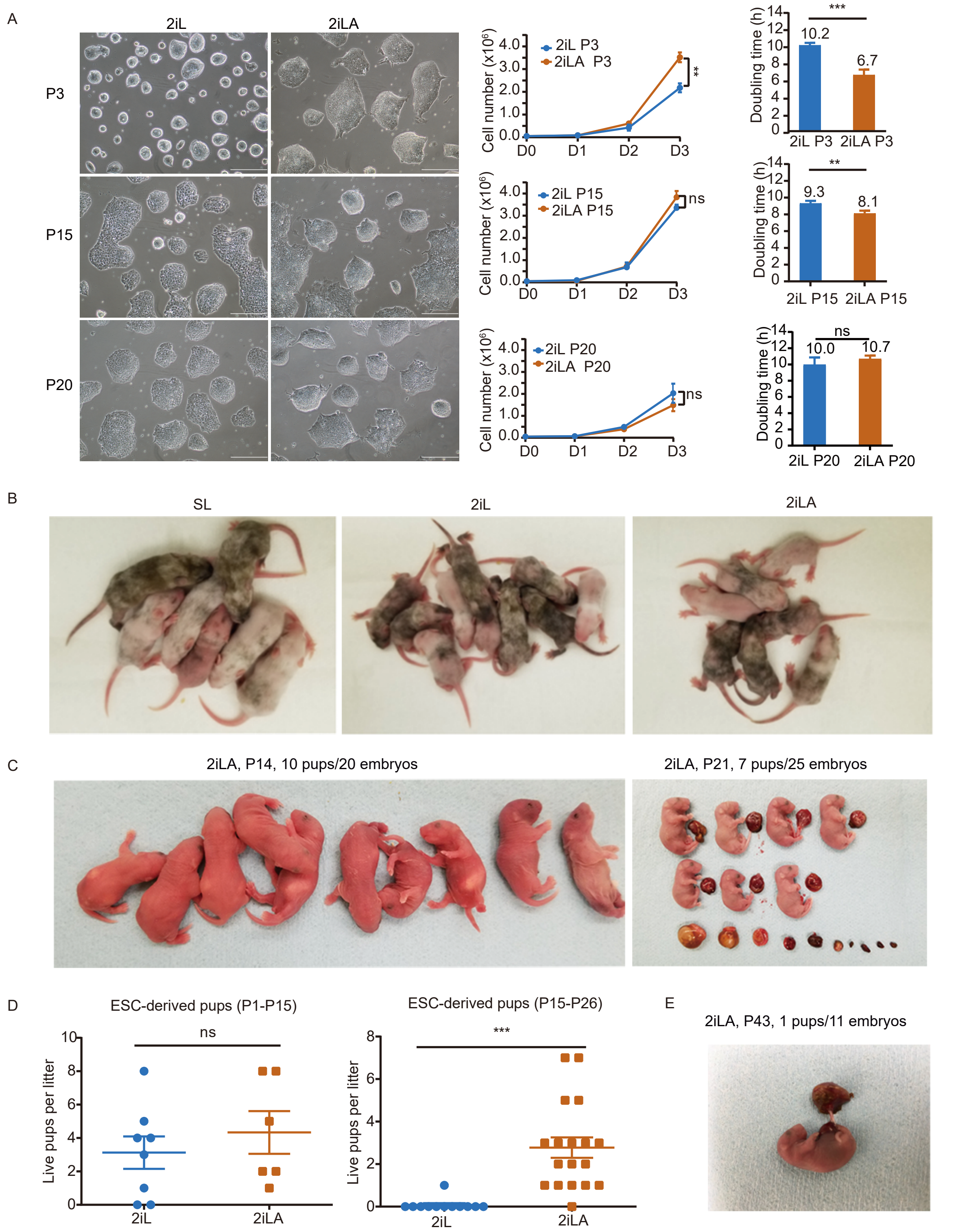

Figure S1. AX promotes ESC proliferation and developmental potency. (A)Representative colonies, cell growth curves and doubling times of ESCs cultured in 2il or 2iLA at P3, 15, and P20. Scale bar in panel a: 25 μm. Replicates (n= 3), ns: no significant difference, *P<0.05, **P<0.01, ***P<0.001, student t-test. (B) Representative chimeras derived from ESCs cultured in 2iL, 2iLA, or SL (serum +LIF) at P3. (C) All-ESC pups from ESCs (ESC-1) cultured in 2iLA at P14 and P21. (D) Dot plots quantifying the all-ESC pups obtained from ESCs (ESC-1) cultured in 2iL or 2iLA. (E) one all-ESC pup obtained from the ESCs (ESC-1) cultured in 2iLA for 43 passages, which is almost three months in vitro.

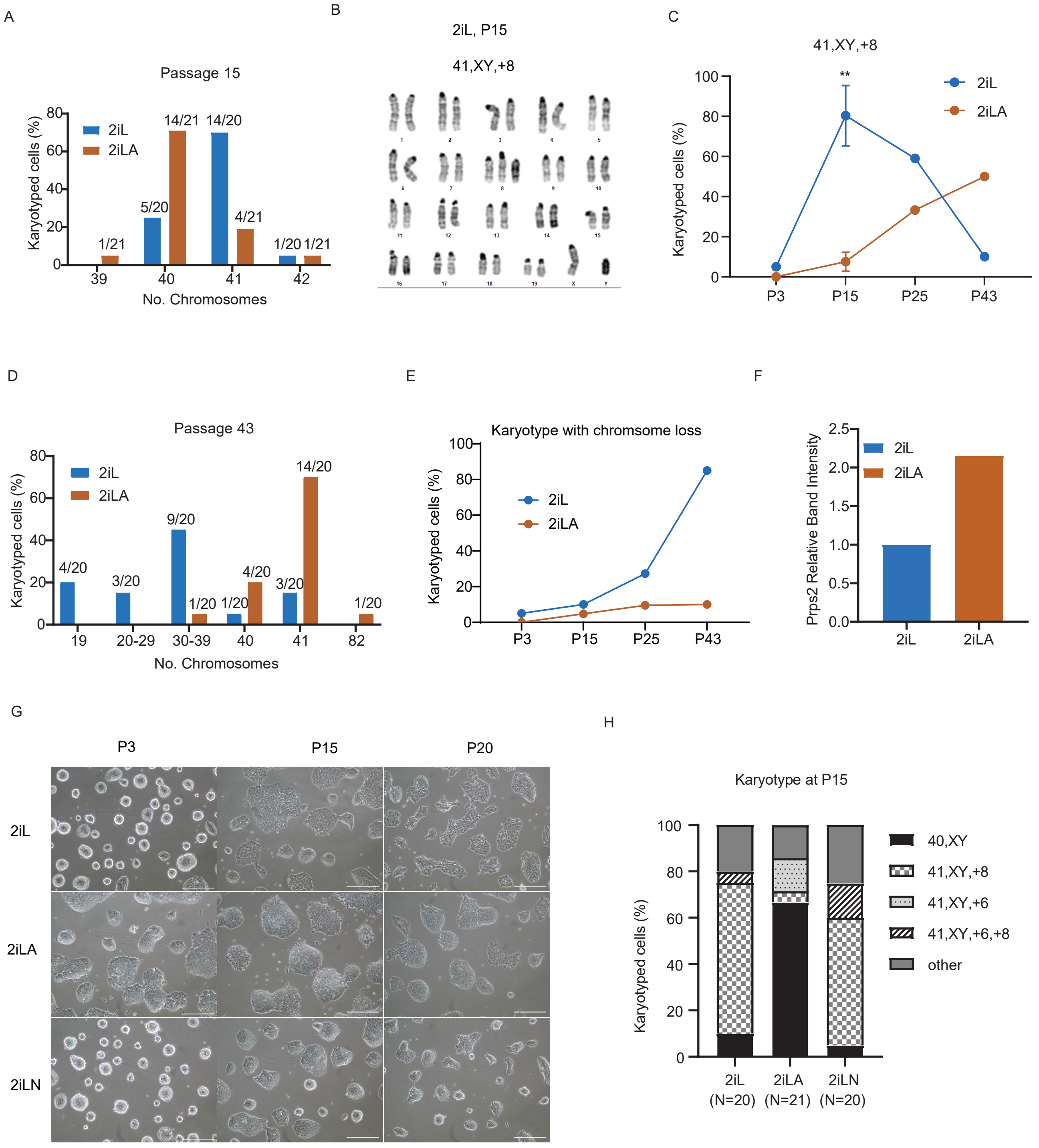

Figure S2. AX improve karyotypic stability of mouse ESCs. (A) Karyotyping analyses for the number of chromosomes of the ESCs cultured in 2il and 2iLA at P15. (B) Representative metaphase spread with trisomy 8 (41, XY, +8) from ESCs in 2iL at P15. Arrow: Trisomy 8. (C) Karyotyping analysis for trisomy 8 in 2iL or 2iLA at different passages (n=~20 metaphases at each passage for 2iL or 2iLA). (D) Karyotyping analyses for the number of chromosomes of the ESCs cultured in 2il and 2iLA at P43. (E) Analysis for karyotype with chromosome loss in 2iL and 2iLA at different passages (n=~20 metaphases at each passage for 2iL or 2iLA). (F) Prps2 relative intensity to ACTIN of western blot bands in 2iL and 2iLA quantified using ImageJ. (G) Colony morphology of ESCs cultured in 2iL, 2iLA, and 2iLN (2iL supplemented with nucleosides) at different passages. Note the colony morphology of ESCs cultured in 2iL at P20 become flat and lost typical dome-shaped morphology, while ESCs in both 2iLA and 2iLN maintained dome-shaped colony morphology. (H) Karyotyping of ESCs cultured in 2iL, 2iLA, and 2iLN at P15.

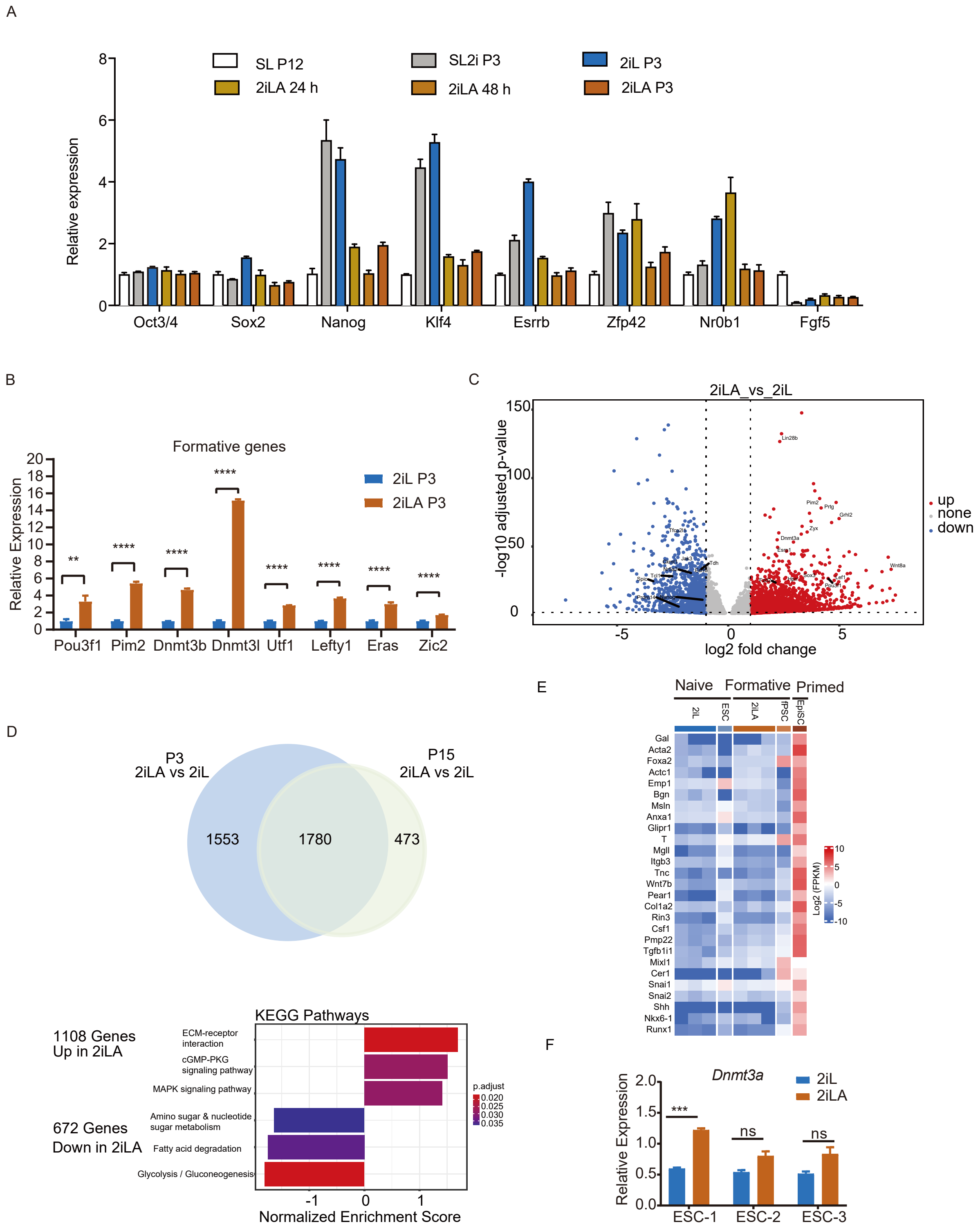

Figure S3. AX induces pluripotency transition. (A)-(B), qRT-PCR for the naive or formative genes for ESCs cultured in 2iL, 2iLA, SL, or SL2i (a) or the formative genes in 2iL or 2iLA at P3 (b). (C) Volcano plot showing fold changes for differentially expressed genes (DEGs) between 2iL and 2iLA at P3. Blue, upregulated in 2iL; Red, upregulated in 2iLA. (D) Venn diagram plot of differentially expressed genes (DEGs) between 2iL and 2iLA ESCs at p3 or p15. Overlapping DEGs with FDR<0.1 & abs (FC)>1 between P3 and P15 and GO analysis for DEGs from bulk RNA-seq. n= three biological replicates for RNA-seq. (E) Gene expression heatmap of primed genes, the mESC (Naive), fPSC (Formative), and EpiSC (Primed) are from published data (Wang et al., 2021). (F) Dnmt3a expression in 2iL or2iLA of three ESC lines.

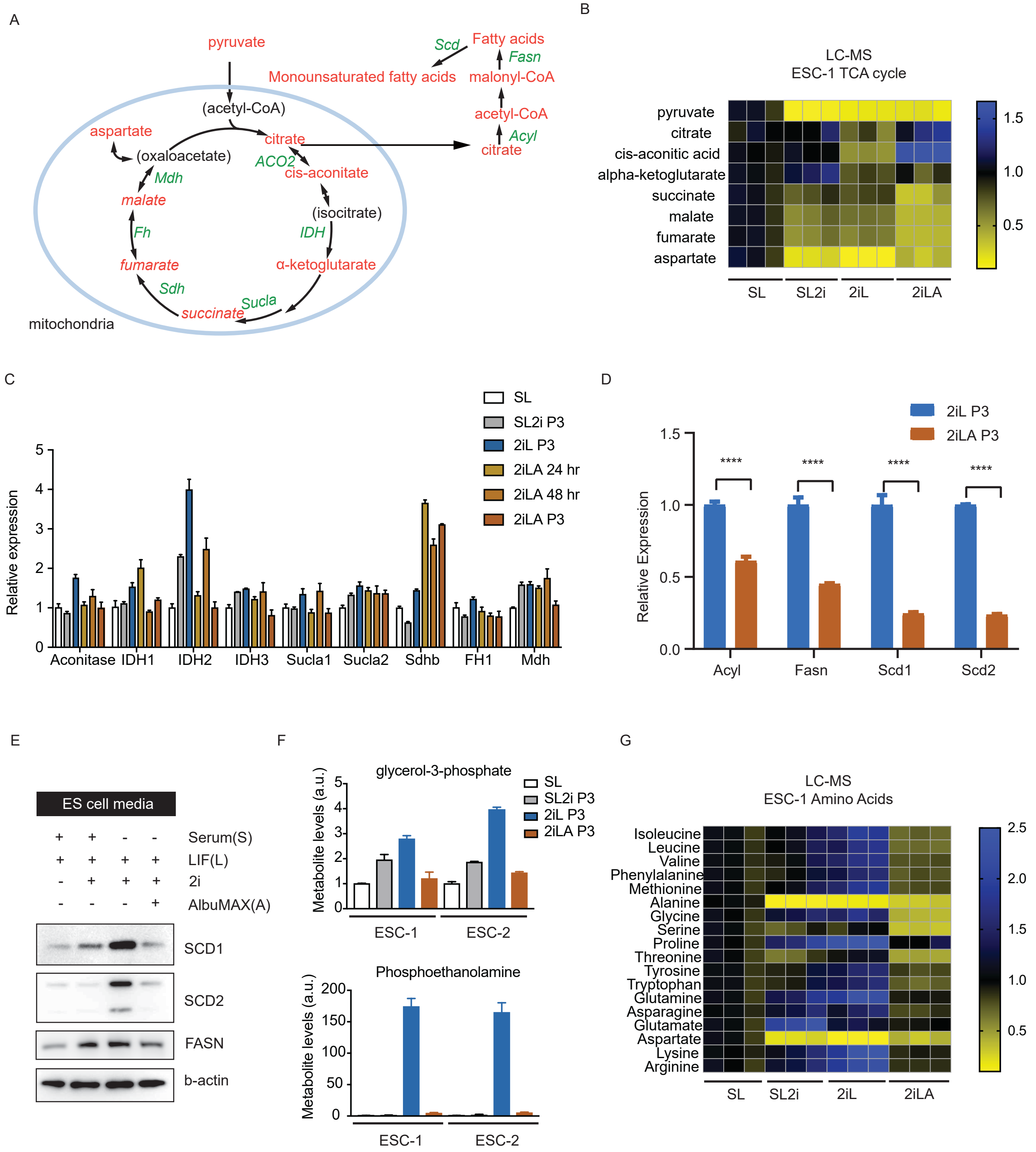

Figure S4. Lipids in AlbuMAX impact intracellular metabolism. (A) Schematic of the tricarboxylic acid (TCA) cycle and de novo lipogenesis. (B) LC-MS analysis of TCA cycle for ESCs cultured in SL, SL2i, 2iL or 2iLA. (C) qRT-PCR for TCA related enzyme genes in SL, SL2i, 2iL, 2iLA 24hr, 2iLA 48hr, or 2iLA P3. (D)-(E) qRT-PCR (D) or western blot (E) for de novo lipogenesis related enzymes for ESCs in 2iL or 2iLA at P3. (F) LC-MS analysis of the metabolites glycerol-3-phosphate or phosphoethanolamine for ESCs in SL, SL2i, 2iL or 2iLA. (G) LC-MS analysis of amino acids for ESCs cultured in SL, SL2i, 2iL or 2iLA.

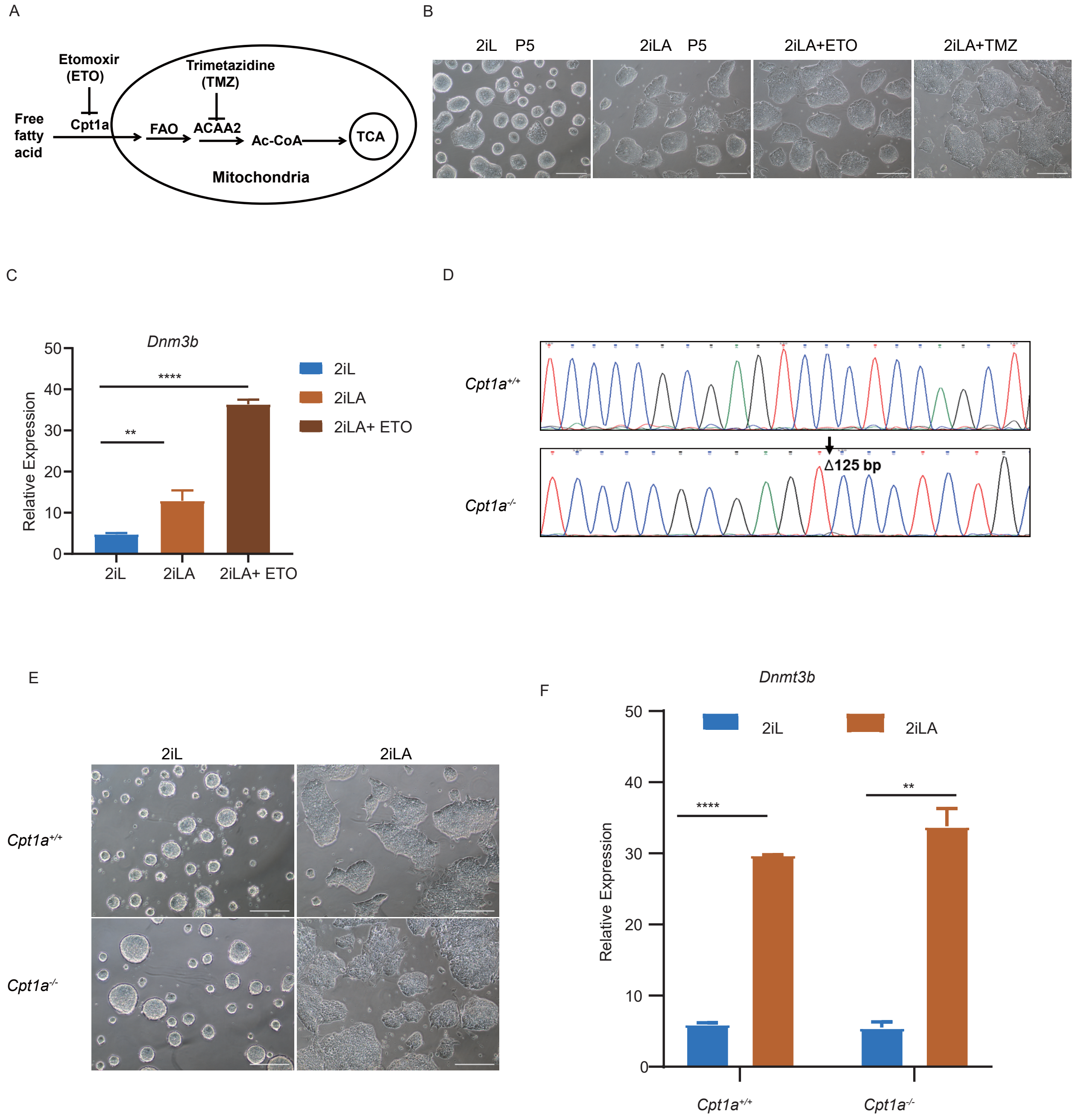

Figure S5. Fatty acid oxidation (FAO) is dispensable for lipid induced pluripotency transition. (A) Schematic illustration of the experimental design for FAO pathway inhibition. (B) Colony morphology after FAO pathway inhibition. Etomoxir (ETO, 100μM), Trimetazidine (TMZ, 200μM). (C) Formative gene Dnmt3b expression after FAO pathway inhibition. Etomoxir (ETO, 100μM), Trimetazidine (TMZ, 200μM). (D) Carnitine Palmitoyltransferase 1A (Cpt1a) deletion in mESCs, homologous deletion (the deletion region 125 bp spanning between exon3 and intron 3). (E) Colony morphology after Cpt1a deletion. (F) Formative gene Dnmt3b expression (qRT-PCR) after Cpt1a deletion.

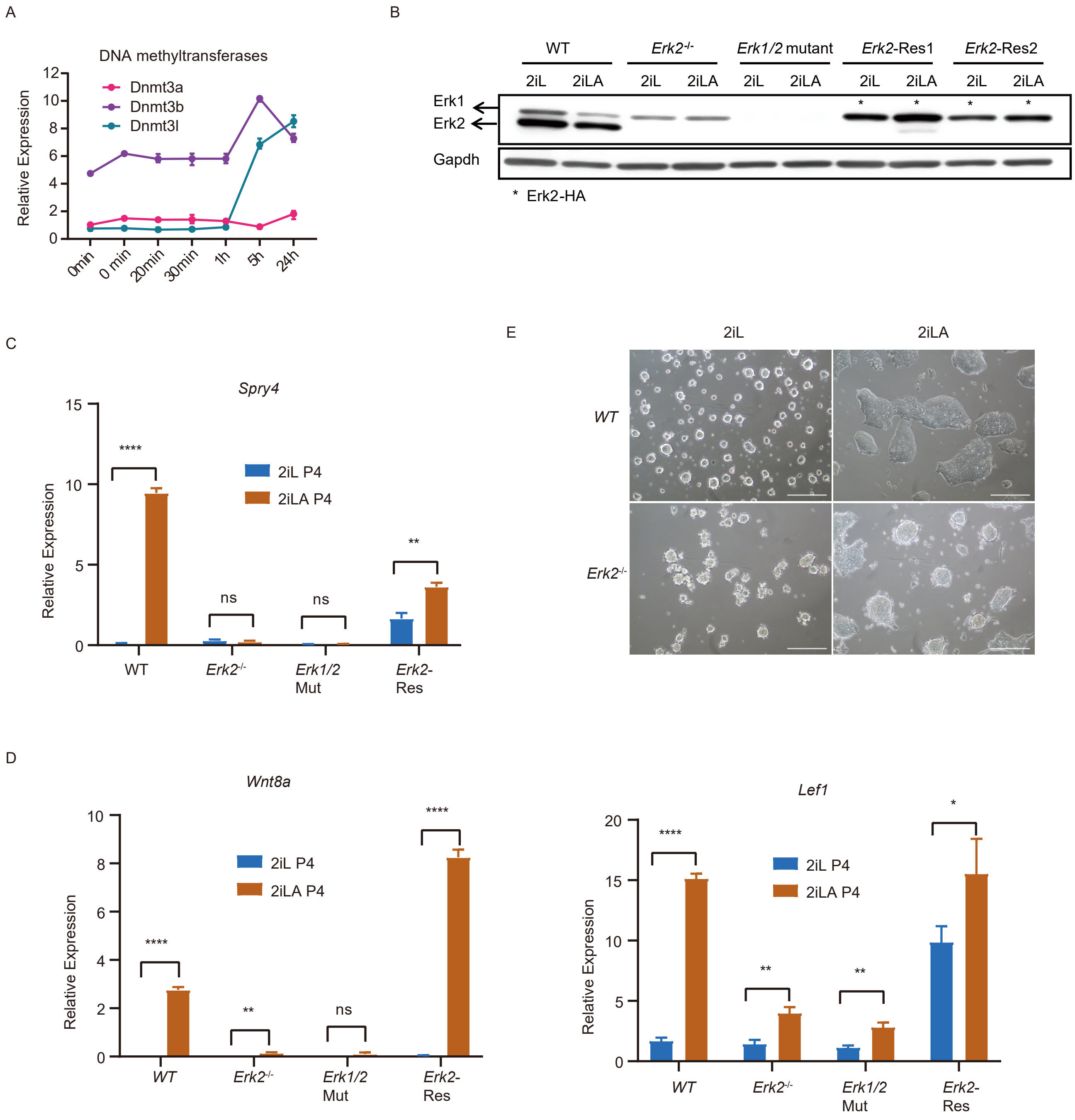

Figure S6. Erk signaling is essential for lipid-induced pluripotency transition. (A) qRT-PCR for Dnmts transcript levels in ESCs after switching 2iL to 2iLA at different time points. (B) Western blotting for Erk1/2 in WT, Erk2-/-, Erk1/2 mutant, Erk2-Res1 or Erk2-Res2 ESCs in 2iL or 2iLA. Erk2-Res: Erk2-rescued in the Erk1/2 mutant ESCs (Lentiviral-based shRNA against Erk1 in the Erk2-/- ESCs). (C)-(D) qRT-PCR for the Erk1/2 target gene Spry4 (C), and the formative marker genes Wnt8a and Lef1 for WT, Erk2-/-, Erk1/2 mutant and Erk2-Res ESCs after 4 passages cultured in 2iL or 2iLA(D). (E) Colony morphology change for WT and Erk2-/- ESCs in 2iL or 2iLA at P3.

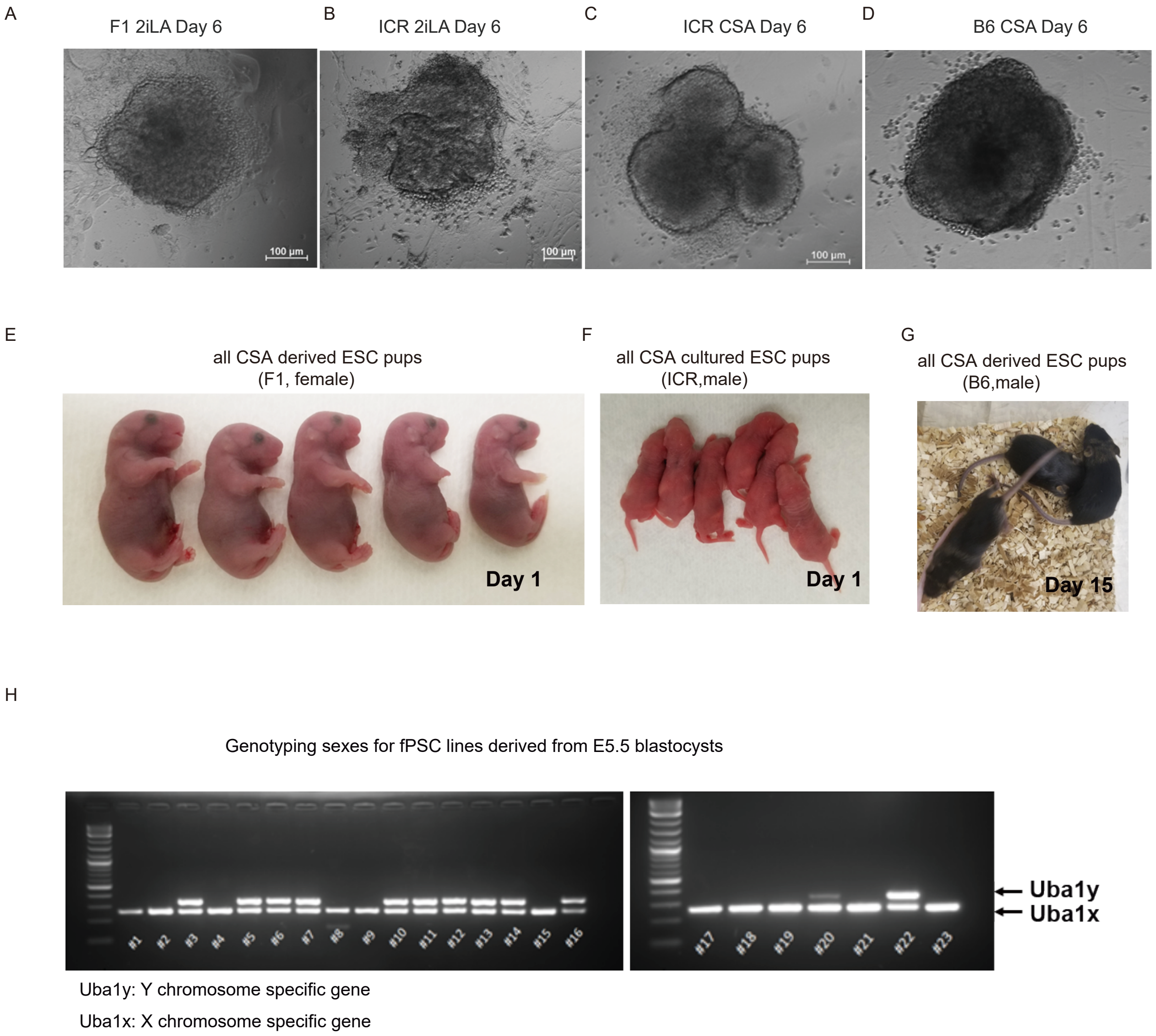

Figure S7. Derivation of ESCs from blastocysts using AX-based media. (A)-(D) The ICM outgrowth from an F1 (129B6), ICR and B6 embryo (E3.5) on feeders at day 6 cultured in 2iLA or CSA medium. (E)-(G) all-ESC pups generated from F1 (E), ICR (F) or B6 (G) ESCs derived from E3.5 blastocysts using CSA medium. (H) Genotyping sexes for the 23 cell lines derived from E5.5 blastocysts using 2iLA medium.

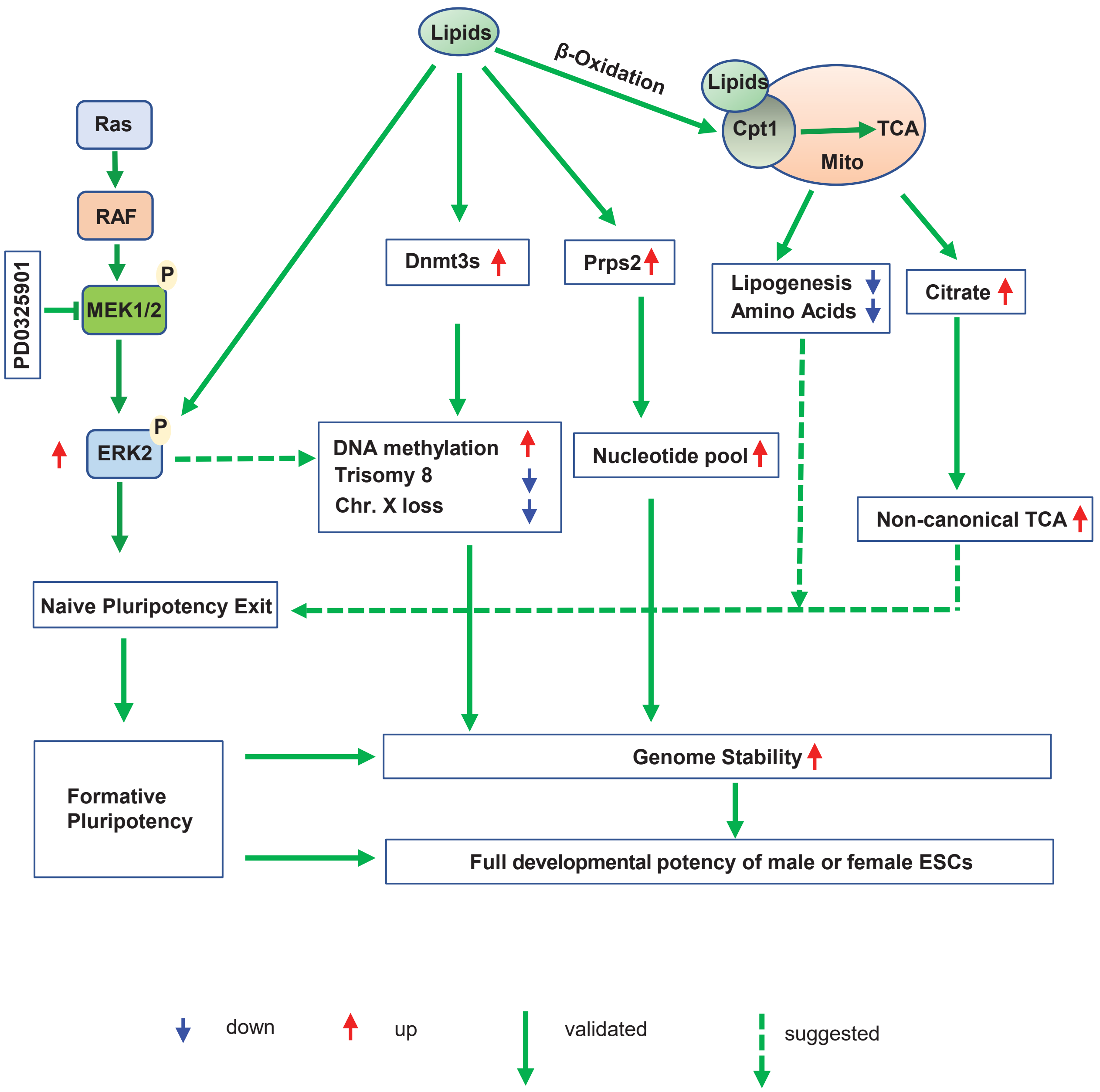

Figure S8. Schematic illustrating the dual role of lipids on genome stability and pluripotency of mouse embryonic stem cells. Lipids directly stimulate Mek-mediated Erk2 phosphorylation which leads to exit of naïve state and establishment of formative pluripotency for ESCs cultured in 2iLA medium. Lipid metabolism reduces the lipogenesis and amino acid biosynthesis and promotes non-canonical TCA metabolites associated with the exit of naïve pluripotency. However, lipid metabolism through β-oxidation is dispensable for transition from naïve to formative state. Lipids enhance DNA methylation and nucleotide pool, therefore promoting genome stability. Stimulation of Erk2 activity by lipids also alleviates X chromosome loss and trisomy 8 for ESCs cultured in 2iLA medium. The dual role of lipids on genome stability and pluripotency facilitates the preservation of full developmental potency of murine ESCs for both sexes during long-term culture in vitro.

References

Akalin, A., Garrett-Bakelman, F.E., Kormaksson, M., Busuttil, J., Zhang, L., Khrebtukova, I., Milne, T.A., Huang, Y., Biswas, D., Hess, J.L., *et al.* (2012). Base-pair resolution DNA methylation sequencing reveals profoundly divergent epigenetic landscapes in acute myeloid leukemia. PLoS Genet *8*, e1002781.

Dodt, M., Roehr, J.T., Ahmed, R., and Dieterich, C. (2012). FLEXBAR-Flexible Barcode and Adapter Processing for Next-Generation Sequencing Platforms. Biology (Basel) *1*, 895-905.

Gu, H., Smith, Z.D., Bock, C., Boyle, P., Gnirke, A., and Meissner, A. (2011). Preparation of reduced representation bisulfite sequencing libraries for genome-scale DNA methylation profiling. Nat Protoc *6*, 468-481.

Krueger, F., and Andrews, S.R. (2011). Bismark: a flexible aligner and methylation caller for Bisulfite-Seq applications. Bioinformatics *27*, 1571-1572.

Langmead, B., and Salzberg, S.L. (2012). Fast gapped-read alignment with Bowtie 2. Nat Methods 9, 357-359.

Wang, X.X., Xiang, Y.L., Yu, Y., Wang, R., Zhang, Y., Xu, Q.H., Sun, H., Zhao, Z.A., Jiang, X.X., Wang, X.Q., et al. (2021). Formative pluripotent stem cells show features of epiblast cells poised for gastrulation. Cell Res 31, 526-541.
